# Targeting Hidden Pathogens: Cell-Penetrating Enzybiotics Eradicate Intracellular Drug-Resistant Staphylococcus aureus

**DOI:** 10.1128/mBio.00209-20

**Published:** 2020-04-14

**Authors:** Christian Röhrig, Markus Huemer, Dominique Lorgé, Samuel Luterbacher, Preeda Phothaworn, Christopher Schefer, Anna M. Sobieraj, Léa V. Zinsli, Srikanth Mairpady Shambat, Nadja Leimer, Anja P. Keller, Fritz Eichenseher, Yang Shen, Sunee Korbsrisate, Annelies S. Zinkernagel, Martin J. Loessner, Mathias Schmelcher

**Affiliations:** a Institute of Food, Nutrition and Health, ETH Zurich, Zurich, Switzerland; b Department of Infectious Diseases and Hospital Epidemiology, University Hospital Zurich, University of Zurich, Zurich, Switzerland; c Department of Immunology, Faculty of Medicine Siriraj Hospital, Mahidol University, Bangkok, Thailand; Institut Pasteur

**Keywords:** endolysin, MRSA, *Staphylococcus aureus*, antibiotic resistance, bacteriophages, cell-penetrating peptide, intracellular bacteria, peptidoglycan hydrolases, persister, protein therapeutic, small-colony variant

## Abstract

Staphylococcus aureus is a major concern in human health care, mostly due to the increasing prevalence of antibiotic resistance. Intracellular localization of S. aureus plays a key role in recurrent infections by protecting the pathogens from antibiotics and immune responses. Peptidoglycan hydrolases (PGHs) are highly specific bactericidal enzymes active against both drug-sensitive and -resistant bacteria. However, PGHs able to effectively target intracellular S. aureus are not yet available. To overcome this limitation, we first screened 322 recombineered PGHs for staphylolytic activity under conditions found inside eukaryotic intracellular compartments. The most active constructs were modified by fusion to different cell-penetrating peptides (CPPs), resulting in increased uptake and enhanced intracellular killing (reduction by up to 4.5 log units) of various S. aureus strains (including methicillin-resistant S. aureus [MRSA]) in different tissue culture infection models. The combined application of synergistic PGH-CPP constructs further enhanced their intracellular efficacy. Finally, synergistically active PGH-CPP cocktails reduced the total S. aureus by more than 2.2 log units in a murine abscess model after peripheral injection. Significantly more intracellular bacteria were killed by the PGH-CPPs than by the PGHs alone. Collectively, our findings show that CPP-fused PGHs are effective novel protein therapeutics against both intracellular and drug-resistant S. aureus.

## Abstract (precis)

The increasing prevalence of antibiotic-resistant bacteria is one of the most urgent problems of our time. Staphylococcus aureus is an important human pathogen that has acquired several mechanisms to evade antibiotic treatment. In addition, S. aureus is able to invade and persist within human cells, hiding from the immune response and antibiotic therapies. For these reasons, novel antibacterial strategies against these pathogens are needed. Here, we developed lytic enzymes which are able to effectively target drug-resistant and intracellular S. aureus. Fusion of these so-called enzybiotics to cell-penetrating peptides enhanced their uptake and intracellular bactericidal activity in cell culture and in an abscess mouse model. Our results suggest that cell-penetrating enzybiotics are a promising new class of therapeutics against staphylococcal infections.

## INTRODUCTION

Staphylococcus aureus is a Gram-positive, opportunistic pathogen which colonizes 30% to 50% of the human population (1). It causes a wide range of diseases, such as bacteremia, endocarditis, osteomyelitis, meningitis, and pneumonia, and it is one of the leading causes of skin and soft tissue infections, such as abscesses (2). S. aureus is able to adapt and survive under various conditions, as it has acquired several mechanisms to evade the host immune response and survive treatment with antibiotics (3). In 2017, methicillin-resistant S. aureus (MRSA) was a leading cause of health care-associated infections in the United States, with 323,700 estimated cases in hospitalized patients and 10,600 MRSA-related deaths (4). The cost associated with each of these infections was estimated to be $35,367 (5). In addition, S. aureus invade eukaryotic host cells and persist and proliferate intracellularly, often resulting in the formation of small-colony variants (SCVs) (6). SCVs can be induced by low pH found in several intracellular compartments or in inflamed tissue. They have reduced metabolic activity and are therefore more tolerant to antibiotic therapy (7). This helps explain the high frequency of recurrent infections observed with S. aureus. Thus, there is the urgent requirement for new antibacterial agents active against intracellular antibiotic-tolerant as well as drug-resistant S. aureus.

Peptidoglycan hydrolases (PGHs) may represent such an alternative antibacterial agent (8). These enzymes cleave specific bonds within the peptidoglycan (PG) of the bacterial cell wall, thereby inducing bacterial lysis. This active killing mechanism also enables killing of metabolically inactive persisters and drug-resistant bacteria (9). The bacteriocin lysostaphin (LST) is produced by Staphylococcus simulans bv. staphylolyticus and specifically targets S. aureus (10). It consists of the following two functional domains: one N-terminal enzymatically active domain (EAD), which belongs to the M23 endopeptidase family and cleaves the pentaglycine bridge within the PG network of S. aureus, and one C-terminal SH3b-type cell wall-binding domain (CBD), which recognizes and binds the PG (11). Endolysins are bacteriophage-derived PGHs, which cause lysis of infected host bacteria from within and liberation of progeny viruses at the end of the phage lytic cycle. When applied from the outside, they rapidly and effectively kill Gram-positive bacteria by degrading the externally exposed PG; for this reason, they are deemed a promising new class of antimicrobials (12). Their major advantages include high specificity for their target bacteria, nontoxicity to eukaryotic cells, and very low risk of resistance development due to their highly conserved PG target bonds (13).

Endolysins also feature a modular structure consisting of EADs and CBDs which facilitates molecular engineering of these proteins (14, 15). Staphylococcal phage endolysins typically consist of two N-terminal EADs linked to one C-terminal SH3b-like CBD. The most common architecture contains an N‑terminal cysteine, histidine-dependent amidohydrolase/peptidase (CHAP) domain, which cleaves the d-alanine-glycine peptide bond, followed by a centrally located amidase domain, which cleaves the *N*-acetylmuramoyl-l-alanine amide bond within the PG (16). Cleavage of one bond likely results in better accessibility of other bonds within the PG network, explaining the synergy observed for combinations of PGHs with different cleavage sites, such as LST and the endolysin LysK (17). Several *in vivo* studies with PGHs and synergistic combinations thereof have previously demonstrated their therapeutic potential, and the first endolysins are undergoing clinical trials (13, 16). However, despite the aforementioned advantages, most PGHs show very low, if any, efficacy against intracellular S. aureus, which is due to a lack of cell-penetrating properties and/or reduced activity under the conditions encountered within eukaryotic cells (18). Therefore, a first step to address this limitation was to identify PGHs with high activity under these conditions. Previous studies have shown that molecular engineering of PGHs, including the generation of chimeras harboring functional domains from different origins, can yield enzymes with such novel and optimized properties (19–21).

For effective intracellular killing, these active enzymes also need to be transduced into eukaryotic cells. For some proteins, this may be achieved through intrinsic cell-penetrating properties, as has been reported for the streptococcal phage endolysin PlyC (22). For other PGHs without this capability, fusion to cell-penetrating peptides (CPPs) has been suggested as one possible delivery strategy (12, 23). CPPs have been shown to increase the internalization of various cargo molecules into eukaryotic cells (24). In a previous study, we observed only moderate intracellular killing efficacies for CPP-tagged LST and a chimeric PGH in multiple cell culture infection models (<1-log reduction compared to the parental enzymes) (21). This suggests that effective intracellular killing depends on other factors besides intracellular delivery, such as finding optimal combinations of highly active PGHs and compatible CPPs that do not impair the lytic activity of the PGH in a fusion construct. Here, we screened a comprehensive PGH library for enzymes with high activity under conditions found in different intracellular compartments, fused the top candidates to selected CPPs, and tested their intracellular activity against S. aureus strains in multiple eukaryotic infection models, individually and in synergistic combinations. The two most promising PGH cocktails were then evaluated in a murine abscess model.

## RESULTS

### Screening identifies PGHs with activity under extra- and intracellular conditions.

To develop effective antimicrobials for the treatment of S. aureus infections involving intracellular and drug-resistant bacteria, we selected 322 PGH constructs with presumptive staphylolytic activity from an extensive library and assessed them in a microtiter plate-based screening assay (20) under conditions simulating relevant physiological settings. Each construct was rated based on its ability to eradicate or reduce S. aureus numbers under extracellular (phosphate-buffered saline [PBS] and Dulbecco’s modified Eagle’s medium [DMEM]), cytosolic (intracellular buffer), and lysosomal (lysosomal buffer) conditions. This initial screening yielded a selection of 36 PGH constructs (approximately 11% of the entire library) that exceeded a relative activity score of 0.6, displaying robust staphylolytic activity throughout the tested conditions (Fig. S1 in the supplemental material). These enzymes represent a diverse collection of PGHs with regard to their EADs and modular architectures and include functional domains from multiple origins, such as the endolysins of phages Twort (Tw) (25), 2638a (26), K (27), SEP1 (SEP) (28), GH15 (29), phi11 (f11) (30), and H5 (31), in addition to the bacteriocins LST and ALE1 (32). Despite its low pH of 4.7, many PGH constructs featuring the CHAP domain of the Tw endolysin (CHAPTw), as well as the construct CHAPSEP_SH3b2638a, displayed high activity in lysosomal buffer. On the other hand, most of the constructs with the M23 domain from LST were highly active in PBS, DMEM, and intracellular buffer. Overall, M23LST was the EAD most frequently represented within the selection, being present in 22 of the 36 PGHs.

10.1128/mBio.00209-20.1 FIG S1 Relative activity scores of the 36 most potent staphylolytic PGHs identified by microtiter plate-based screening simulating extra- and intracellular conditions. Composite scores are the sum of individual scores derived from activities in 4 different buffers or media. Constructs are named according to their domain structure and origin of individual domains. CHAP, CHAP endopeptidase domain; M23, M23 endopeptidase domain; Pep, endolysin-derived endopeptidase domain; Ami, *N*-acetylmuramoyl-l-alanine amidase domain; SH3b, Src-homology 3b domain; Tw, phage Twort endolysin; 2638a, phage 2638a endolysin; K, phage K endolysin (LysK); SEP, phage SEP1 endolysin; f11, phage phi11 endolysin; GH15, phage GH15 endolysin; LysH5, phage H5 endolysin; ALE1, bacteriocin ALE1; LST, lysostaphin; Xa, factor Xa protease cleavage site; TEV, tobacco etch virus cleavage site; PTD, protein transduction domain; H, His tag. Download FIG S1, PDF file, 0.2 MB. Copyright © 2020 Röhrig et al. 2020 Röhrig et al. This content is distributed under the terms of the Creative Commons Attribution 4.0 International license.

### Selected PGHs display high staphylolytic activity in multiple assays.

**FIG 1:**
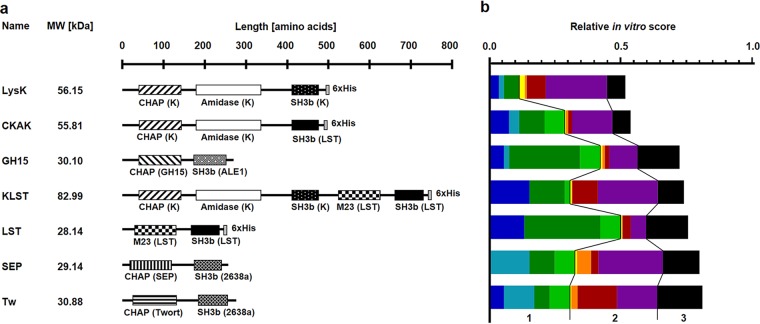
Modular architectures and relative *in vitro* activity scores of selected PGHs. (a and b) Shown are the names, molecular weights (MW), domain structures, and lengths in amino acids of the seven PGHs most active against S. aureus (a) based on their relative *in vitro* scores composed of the weighted scores of the three activity assays, two of which were carried out under the following extra- and intracellular conditions: (i) time-kill assay (TKA) in PBS (blue), lysosomal buffer (turquoise), DMEM, (dark green), and intracellular buffer (light green); (ii) turbidity reduction assay (TRA) in PBS (yellow), lysosomal buffer (orange), DMEM (red), and intracellular buffer (purple); and (iii) spot on the lawn assay (SLA) (black) (b). Origins of individual functional domains are provided in parentheses. CHAP, CHAP endopeptidase domain; M23, M23 endopeptidase domain; amidase, *N*-acetylmuramoyl-l-alanine amidase domain; SH3b, Src-homology 3b domain; Twort, phage Twort endolysin; 2638a, phage 2638a endolysin; K, phage K endolysin (LysK); SEP, phage SEP1 endolysin; GH15, phage GH15 endolysin; ALE1, bacteriocin ALE1; LST, lysostaphin; 6×His, His tag.

We expressed and purified the 36 PGHs identified by our screening approach and quantitatively compared them in three different *in vitro* activity assays, namely, a time kill assay (TKA), turbidity reduction assay (TRA), and spot-on-lawn assay (SLA) (all reviewed in references 8 and 12). To gain deeper insight into the unique characteristics of each individual construct, TKAs and TRAs were performed in the four different buffers previously used in the microplate-based screening. Based on the cumulative scores derived from the results of these assays, we ranked the candidates and excluded PGH constructs with obvious drawbacks, such as a lack of antibacterial activity in TKAs. The seven constructs with the highest overall score included the 4 single-EAD constructs CHAPGH15_SH3bALE1 (GH15), LST_H (LST), CHAPSEP_SH3b2638a (SEP), and CHAPTw_SH3b2638a (Tw), as well as 3 constructs harboring multiple EADs, namely, LysK_H (LysK), CHAPK_AmiK_SH3bLST_H (CKAK), and LysK_LST_H (KLST) (Fig. 1a). Of note, this selection of PGHs includes enzymes with unique properties that may be of relevance for the treatment of S. aureus infections. For example, SEP and Tw displayed high activity at low pH (consistent with the results of the screening) (Fig. 1b), which may be beneficial not only when targeting bacteria residing in intracellular phagolysosomes but also in other acidified environments, such as inflamed tissue or abscesses. Furthermore, the smaller single-EAD enzymes generally showed higher activity in SLAs, which can be attributed to their faster diffusion through the semisolid agar matrix (12), a property that may also be of interest for certain scenarios where effective diffusion through tissues is desired. We next aimed at enhancing the translocation of the selected enzymes into eukaryotic cells by fusion to CPPs for effective killing of intracellular S. aureus.

### PGHs retain high activity upon fusion to TAT.

**FIG 2:**
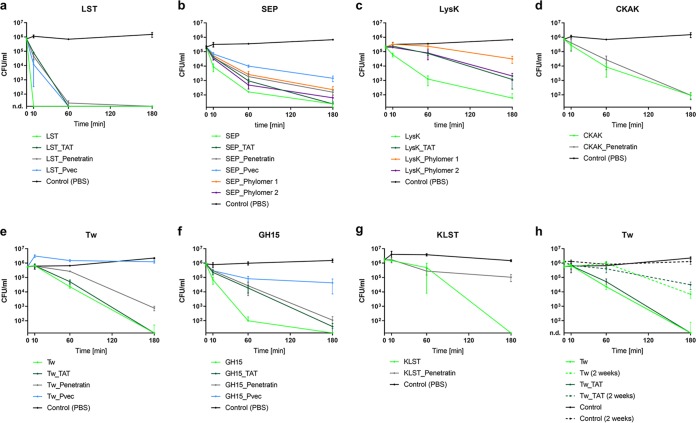
(a to h) Time kill assays with CPP-fused and parental PGHs. Intracellular buffer was spiked with approximately 10^6^ CFU/ml S. aureus Newman. The PGH concentration used was 100 nM. (h) Dotted lines represent the activity after storage of PGHs on ice at 4°C for 2 weeks. Values represent the mean ± standard error of the mean (SEM) of the results from three independent experiments (*n* = 3).

The seven selected enzymes (here named “parental PGHs”) were fused to various CPPs (Table 1), cloned, expressed in Escherichia coli, and purified. At this stage, His tag-free versions of all constructs were used to avoid possible biases. All proteins containing the CPP KalaSyn, as well as LysK, KLST, and CKAK fused to certain CPPs such as Pvec, were found to be either insoluble or insufficiently expressed and were therefore excluded from further analysis. The *in vitro* antibacterial activities of the remaining PGH-CPP fusion proteins were compared with those of the respective parental enzymes (Fig. 2). In general, PGH activities were reduced upon fusion to CPPs, albeit the severity of this effect was dependent on the type of CPP and the enzyme used. The CPP Trans-Activator of Transcription (TAT) generally had the least detrimental effect, with Tw_TAT and SEP_TAT retaining almost the same level of activity as the respective parental enzymes. In contrast, Pvec had the most detrimental impact and attenuated the activities of all PGHs. Tw and its CPP-containing variants were found to suffer from low stability during storage and a consequent decline in activity (Fig. 2h). Therefore, these constructs were excluded from further analysis.

**TABLE 1:** Cell-penetrating peptides evaluated in this study

Peptide	Amino acid sequence	Reference
TAT	GRKKRRQRRRPPQ	79
Penetratin (AntpHD43-58)	RQIKIWFQNRRMKWKK	80
KalaSyn	WEAKLAKALAKALAKHLAKALAKALKACEA	81
Pvec	LLIILRRRIRKQAHAHSK	82
Phylomer 1	RFRCGRRKWQIGS	83
Phylomer 2	WTISSRRRKVNRAC	83

### TAT enhances uptake of PGHs into eukaryotic cells.

**FIG 3:**
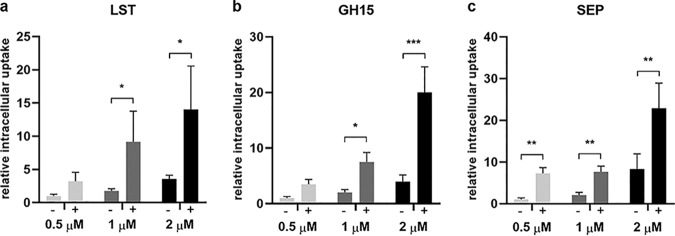
Uptake of parental and CPP‑fused PGHs into eukaryotic cells. Relative uptake of parental (−) and TAT-fused (+) PGHs into A549 cells was quantified by time-resolved fluorescence of DTBTA-Eu^3+^ conjugates. Signals are normalized to the parental PGHs at 0.5 μM. (a to c) Values represent the mean ± SEM of the results from six independent experiments (*n* = 6) for LST (a) and three independent experiments (*n* = 3) for GH15 (b) and SEP (c). Asterisks (*) indicate levels of significance (*, *P* ≤ 0.05; **, *P* ≤ 0.01; ***, *P* ≤ 0.001).

To elucidate whether TAT increases the uptake of our selected PGHs into eukaryotic cells, we conjugated LST, GH15, SEP, and their TAT-containing derivatives to the europium-charged chelator {{2,2′,2″,2‴-{4′-{[(4,6-dichloro-1,3,5-triazin-2-yl)amino]biphenyl-4-yl}-2,2′:6′,2″-terpyridine-6,6′′-diyl}bis-(methylenenitrilo)}tetrakis(acetato)} europium(III) (DTBTA-Eu^3+^). When A549 epithelial cells were incubated with different concentrations of europium-labeled PGHs for 4 h, we observed a dose-dependent uptake of all proteins. Notably, a higher uptake rate was observed for the TAT-fused versions of all three proteins than that for the parental PGHs (Fig. 3). These results corroborate previous findings suggesting that TAT can increase the intracellular uptake of PGHs (21). Furthermore, our data also suggest that some parental, unmodified PGHs may translocate into eukaryotic cells in a dose-dependent manner, albeit at a significantly lower rate than with a TAT.

### CPP-fused lysostaphin eliminates intracellular S. aureus.

To test whether the increased cellular uptake of CPP-fused PGHs translates into enhanced killing of intracellular S. aureus, we determined the intracellular killing efficacies of all parental and CPP-fused PGHs in three different eukaryotic cell lines (A549, MG-63, and 3T3-L1) infected with three different S. aureus strains (Newman, Cowan, and USA300 JE2). We screened for killing of S. aureus in this broad selection of cell lines to find PGH-CPP constructs with the ability to kill intracellular S. aureus regardless of the host cell tissue type. The selected cell lines originate from different tissues/organs within the body, which can all be infected by S. aureus, namely, lung, bone, and connective tissue.

**FIG 4:**
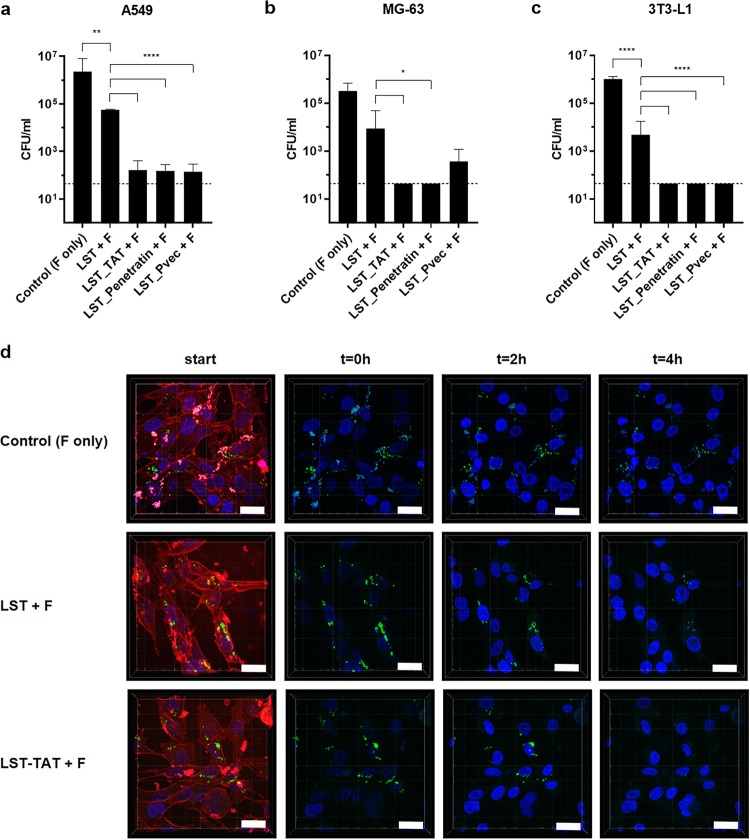
Killing of intracellular S. aureus by LST and its CPP-containing derivatives. (a to c) A549 (a), MG63 (b), and 3T3-L1 cells (c) were infected with S. aureus Cowan (MOI, 1) for 3 h and treated with 2 μM LST or LST-CPP fusions for 4 h. One mg/ml flucloxacillin (F) was present in the medium for the duration of the entire experiment. Values represent means ± SEM of the results from three independent experiments. Asterisks (*) indicate levels of significance (*, *P* ≤ 0.05; **, *P* ≤ 0.01; ****, *P* ≤ 0.0001). (d) CLSM images of MG-63 cells infected with S. aureus RN9623 (green) and exposed to PBS (control), LST, or LST-TAT for 0, 2, and 4 h. DNA was stained with Hoechst 33342 (blue) and cell membranes with FM464 (red). Images with the red channel are shown at the initiation of treatment to confirm cell integrity and intracellular localization of S. aureus. Scale bar = 30 μm.

After infection, residual extracellular S. aureus cells were inactivated by the addition of flucloxacillin before treatment with the PGHs. When infected cells were exposed to LST and its CPP-containing derivatives for 4 h, we consistently found the intracellular killing activity of the CPP-fused variants to be significantly higher than that of the parental enzyme (Fig. 4a to c; see also Table S1 in the supplemental material). While parental LST reduced intracellular bacterial numbers by up to 2.5 log units, complete eradication of S. aureus (numbers below the detection limit) was achieved with CPP-fused LST variants in the majority of the cell line-strain combinations (Table S1). The most effective construct was LST_TAT, which caused eradication of detectable intracellular staphylococci in 8 out of 9 cases. Although LST_Pvec was slightly less effective than were other CPPs, it was still superior to parental LST in all but one case, despite its lower *in vitro* efficacy (Fig. 2), suggesting effective intracellular transduction.

10.1128/mBio.00209-20.4 TABLE S1 Killing of intracellular S. aureus strains by PGH and PGH-CPP constructs in three eukaryotic cell lines after exposure for 4 h. Download Table S1, PDF file, 0.3 MB. Copyright © 2020 Röhrig et al. 2020 Röhrig et al. This content is distributed under the terms of the Creative Commons Attribution 4.0 International license.

To visualize the lysis of intracellular S. aureus, MG-63 cells infected with a green fluorescent protein (GFP)-expressing S. aureus strain were exposed to LST, LST_TAT, or PBS and monitored by confocal laser scanning microscopy (CLSM). In these experiments, both LST and LST-TAT caused a reduction in the number of intracellular S. aureus bacteria compared to the control (Fig. 4d and Movies S1 to S3). After 2 h, only minor differences in bacterial numbers were observed between LST and LST_TAT. However, after 4 h, S. aureus numbers were largely reduced in the LST_TAT treatment compared to the LST treatment. Notably, the GFP signal of destroyed bacterial cells disappeared from one image frame to the next (representing a difference of 5 min). This points toward a rapid release of cytosolic GFP upon lysis of S. aureus and is in agreement with the active killing mechanism of PGHs.

10.1128/mBio.00209-20.8 MOVIE S1 Time-resolved three-dimensional CLSM video of eukaryotic cells infected with S. aureus and treated with PBS (control treatment). The GFP-expressing S. aureus RN9623 (green) was used for infection of MG-63 cells, and flucloxacillin was added to the medium after infection. DNA was stained with Hoechst 33342 (blue) and cell membranes with FM464 (red). Treatment with PBS was initiated at *t* = 0 h. To demonstrate intracellular localization of S. aureus, the red channel was imaged at *t* = 0 h but not used afterwards to minimize additional photobleaching. z-stacks were recorded every 5 minutes for 5 h. Scale bar = 30 μm. Download Movie S1, AVI file, 8.4 MB. Copyright © 2020 Röhrig et al. 2020 Röhrig et al. This content is distributed under the terms of the Creative Commons Attribution 4.0 International license.

### Engineered phage endolysins effectively kill intracellular S. aureus.

The phage endolysin-derived PGHs within our selection and their CPP-fused variants also showed robust killing of intracellular S. aureus in the coculture models. The effects were dependent on the eukaryotic cell line used, with the most significant reductions in intracellular CFU observed in the 3T3-L1 adipocytes (Table S1).

**FIG 5:**
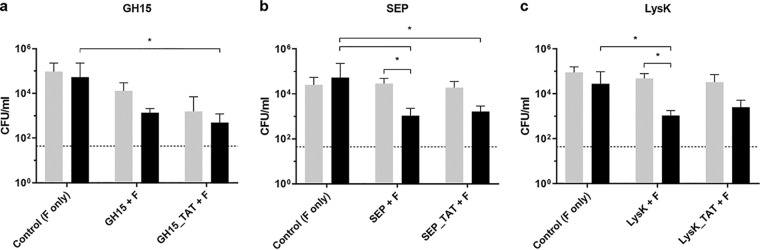
Killing of intracellular MRSA by engineered endolysins and their TAT-fused derivatives. (a to c) MG63 cells infected with MRSA USA300 JE2 (MOI, 1) for 3 h were treated with 2 μM GH15 or GH15-TAT (a), SEP or SEP-TAT (b), and LysK or LysK-TAT (c) for 4 h (light gray) and 15 h (black). One milligram per milliliter flucloxacillin (F) was present in the medium for the entire duration of the experiment. Values represent the mean ± SEM of the results from three independent experiments. Asterisks (*) indicate levels of significance (*, *P* ≤ 0.05).

In an effort to further enhance intracellular killing efficacy in other cell lines, we increased the incubation time in our infection model from 4 to 15 h for a selection of three parental PGHs (GH15, SEP, and LysK) and their TAT-containing derivatives. For all three PGHs, increased killing in MG-63 cells was observed after 15 h, with GH15-TAT, SEP, SEP-TAT, and LysK significantly reducing intracellular S. aureus compared to the control (Fig. 5). It is worth noting that, in contrast to LST, killing of intracellular bacteria by endolysin-derived PGHs was largely independent of the presence of a CPP, suggesting considerable intrinsic internalization propensities of these enzymes.

### CPP-fused PGHs act synergistically to kill intracellular S. aureus.

**FIG 6:**
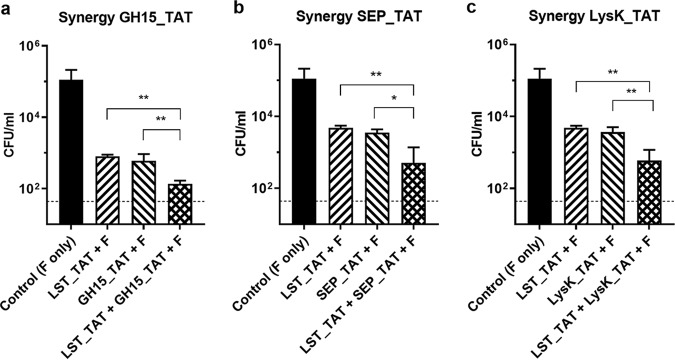
Intracellular synergy of CPP-fused PGHs against MRSA. MG63 cells infected with S. aureus USA300 JE2 (MOI, 1) for 3 h were exposed to individual CPP-fused PGHs and combinations thereof for 15 h. (a to c) The concentrations of single PGHs were 0.04 μM for LST_TAT and 4 μM GH15_ TAT (a), 0.01 μM for LST_TAT and 1 μM for SEP_TAT (b), and 0.02 μM for LST_TAT and 2 μM for LysK_TAT (c). The concentrations of each component in the mixtures were half of those used for the individual treatments. One milligram per milliliter flucloxacillin (F) was present in the medium for the entire duration of the experiment. Values represent the mean ± SEM of the results from three independent experiments. Asterisks (*) indicate levels of significance (*, *P* ≤ 0.05; **, *P* ≤ 0.01).

Previous studies have demonstrated that combining PGHs targeting different chemical bonds in the PG, such as those harboring a CHAP domain and an M23 endopeptidase domain, makes it possible to capitalize on synergistic antibacterial effects (17, 20). To explore this phenomenon in an intracellular setting, we first defined concentrations for 4 individual enzymes (LST-TAT, GH15-TAT, SEP-TAT, and LysK-TAT) that caused similar reductions in intracellular S. aureus in our coculture model. Subsequently, we compared the efficacies of each tested pair of individual enzymes at these predetermined concentrations to that of a mixture of both enzymes at half the concentrations. In all cases, the PGH mixture killed significantly more bacteria than did the single PGHs (Fig. 6). These results clearly demonstrate intracellular synergy of the tested PGH combinations, highlighting a strategy to maximize intracellular killing.

### CPP-fused PGHs are noncytotoxic.

**FIG 7:**
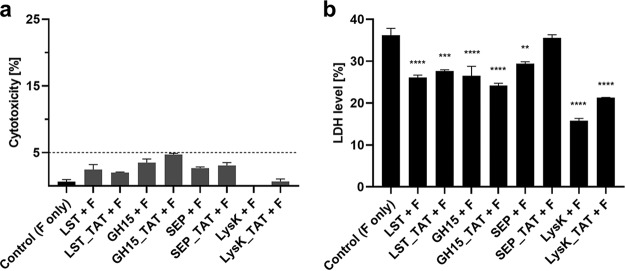
Cytotoxicity and LDH levels in supernatant of MG-63 cells after PGH treatment. (a) Cells were treated with flucloxacillin only or flucloxacillin and parental or CPP-fused PGHs for 15 h. (b) Cells infected with S. aureus USA300 JE2 were treated as described in panel a, and LDH levels were measured. Analysis was performed with the Pierce LDH cytotoxicity assay. One milligram per milliliter flucloxacillin (F) was present in the medium for the entire duration of the experiment. Values represent the mean ± SEM of the results from three independent experiments. Asterisks (*) indicate levels of significance (*, *P* ≤ 0.05; **, *P* ≤ 0.01; ***, *P* ≤ 0.001; ****, *P* ≤ 0.0001).

One important concern when using agents with the ability to translocate into eukaryotic cells is potential cytotoxic effects. To test whether parental and CPP-fused PGHs are harmful to eukaryotic cells, we investigated their cytotoxicity in MG-63 cells. After exposure for 15 h, no cytotoxicity was observed (Fig. 7a). Additionally, we exposed MG-63 cells infected with a MRSA strain of the USA300 lineage (known for its high levels of cytotoxicity [33]) to PBS, PGHs, and CPP-fused PGHs and measured the lactate dehydrogenase (LDH) levels in the supernatant after 15 h of treatment (Fig. 7b). LDH levels of cells treated with PGH or CPP-fused PGHs were significantly lower than those of the PBS control in almost all cases. Since LDH can also originate from bacteria (34), this suggests that either fewer LDH-producing bacteria were present inside the MG-63 cells treated with the PGHs or that there was less cytotoxic damage due to a reduction of intracellular S. aureus bacteria. A combination of both effects could also be possible. In either case, this demonstrates that PGHs and CPP-fused PGHs exert a positive effect on S. aureus-infected cells and are not cytotoxic.

### Synergistic PGH-TAT cocktails effectively kill S. aureus in a murine abscess model.

Using a murine subcutaneous abscess model, we assessed the ability of three of our selected CPP-fused PGHs to treat bacterial infections involving intracellular S. aureus (35). S. aureus Cowan, which has previously demonstrated high invasion rates into cells and is able to persist intracellularly (7), was used for infection, resulting in abscess formation. In a preliminary set of dose-finding experiments, different concentrations of two synergistic mixtures of CPP-fused PGHs, cocktail_1_TAT (GH15_TAT and LST_TAT) and cocktail_2_TAT (SEP_TAT and LST_TAT), were injected into the tissue in close proximity to the abscesses 24, 48, and 72 h after the infection. Additionally, the mice received flucloxacillin to reduce extracellular bacteria. As shown in Fig. S2a to c, both cocktails caused a dose-dependent decrease in total bacterial numbers in the abscess, as well as numbers in the pus and the surrounding tissue, with cocktail_2_TAT being more effective than cocktail_1_TAT. Similar effects were observed for the number of intracellular bacteria in the pus (Fig. S2d). Cocktail_2_TAT was also more effective at alleviating bodyweight loss in the animals than were cocktail_1_TAT and the control (Fig. S2e).

10.1128/mBio.00209-20.2 FIG S2 Efficacy of different doses of cocktail_1_TAT and cocktail_2_TAT against S. aureus Cowan in a murine abscess model. Cocktail_1_TAT was tested at 4 μg (light-pink squares), 20 μg (pink squares), and 100 μg (dark-pink squares), and cocktail_2_TAT was tested at 4 μg (light-blue squares), 20 μg (blue squares), and 100 μg (dark-blue squares). All animals received 1 mg flucloxacillin (F) at days 2 and 3 postinfection. (a to c) Total S. aureus numbers in the abscesses after 4 days (a) were determined as the sum of bacterial numbers in pus (b) and the surrounding tissue (c). (d) Relative numbers of intracellular bacteria in pus after treatment of pus with flucloxacillin *ex vivo*. (e) Relative mean bodyweight of mice treated with cocktail_1_TAT (pink squares), cocktail_2_TAT (blue squares), or buffer (black circles) over the time of infection and treatment. Error bars show the standard error of the mean (SEM). The administration of PGHs and flucloxacillin is indicated by black and gray arrows, respectively. Download FIG S2, PDF file, 0.4 MB. Copyright © 2020 Röhrig et al. 2020 Röhrig et al. This content is distributed under the terms of the Creative Commons Attribution 4.0 International license.

**FIG 8:**
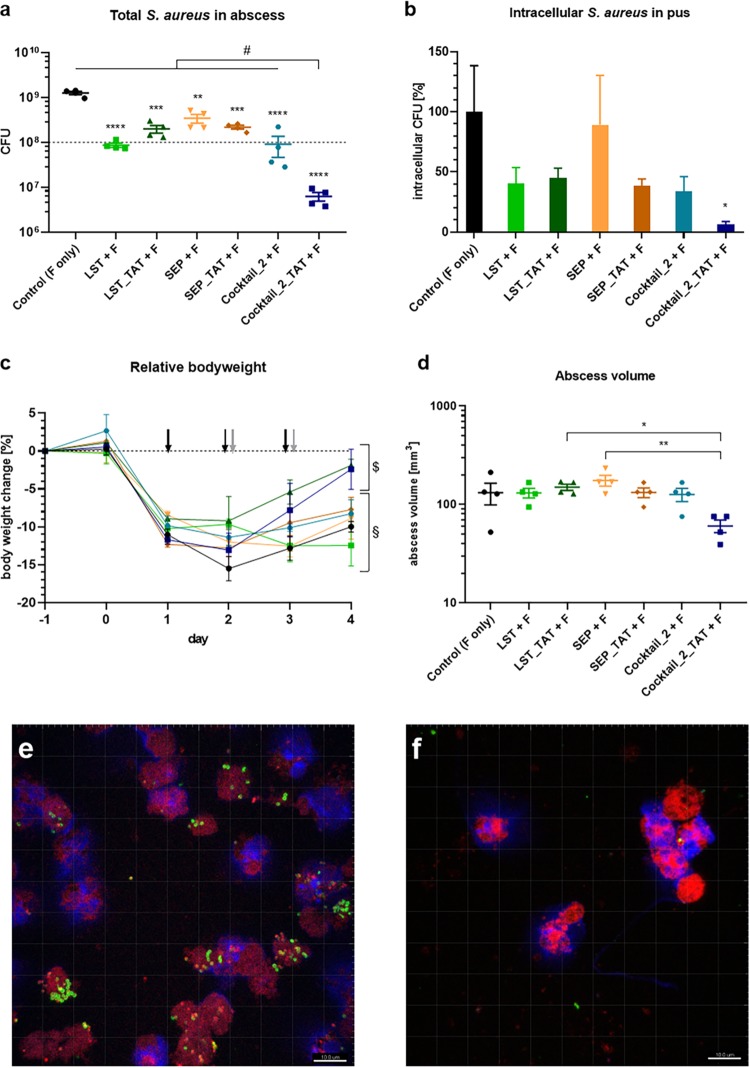
Efficacy of a synergistic PGH cocktail and its individual components against S. aureus in a murine abscess model. Animals infected with S. aureus Cowan were treated with 100 μg of LST (green squares), LST_TAT (dark-green triangles), SEP (orange inverted triangles), SEP_TAT (dark-orange diamonds), cocktail_2 (teal circles), and cocktail_2_TAT (navy-blue squares) on days 1, 2, and 3 after infection. All animals including control mice (black circles) received 1 mg flucloxacillin (F) intraperitoneally (i.p.) at days 2 and 3 after infection. (a and b) The number of viable S. aureus after the different treatments was determined in the whole abscesses (a) and intracellularly within host cells in the pus after additional treatment with flucloxacillin *ex vivo* (b). The pound symbol (#) in panel a indicates a statistically significant difference between cocktail_2_TAT and all other treatments (*P* ≤ 0.0001). (c and d) Additionally, the relative bodyweights of mice over the time of infection and treatment (c) and the volume of the abscesses at the endpoint (d) were determined. In panel c, the time points of PGH and flucloxacillin treatments are indicated with black and gray arrows, respectively. The paragraph symbol (§) indicates statistically significant differences in bodyweight between day 0 and day 4 within the same treatment (*P* ≤ 0.01), whereas the dollar sign ($) indicates no significant differences. (e and f) Representative CLSM image of pus smears from control mice (e) and animals treated with cocktail 2_TAT (f). Values represent the mean ± SEM of the results from four biological replicates for each group. Asterisks (*) indicate levels of significance (*, *P* ≤ 0.05; **, *P* ≤ 0.01; ***, *P* ≤ 0.001; ****, *P* ≤ 0.0001).

We therefore chose cocktail_2_TAT to further analyze its therapeutic potential in the murine abscess model, in comparison with its individual components (LST_TAT and SEP_TAT), as well as the respective parental (i.e., TAT-free) PGHs LST and SEP, alone and in combination (cocktail_2). As shown in Fig. 8a, cocktail_2_TAT caused the highest reduction in total bacterial numbers of all treatments compared to the control (2.30 log units), and similar observations were made for the CFU in the pus and the surrounding tissue (Fig. S3a and b). The increase in viable bacteria in the control from 10^8^ CFU (inoculum) to 1.3 × 10^9^ CFU during the course of the experiment indicates that S. aureus Cowan was able to proliferate in the mice despite flucloxacillin treatment. The synergism of LST_TAT and SEP_TAT that we had demonstrated in the cell culture model was also apparent *in vivo*, since the treatment with the individual PGHs was significantly less effective than with cocktail_2_TAT (Fig. 8a and c). Importantly, cocktail_2_TAT also had a significantly stronger effect than that of cocktail_2, highlighting the added value of the CPP for therapeutic efficacy. Furthermore, cocktail_2_TAT was the only treatment that significantly reduced the number of intracellular S. aureus bacteria within host cells present in the pus (Fig. 8b). This finding was supported by the analysis of CLSM images obtained from pus smears of the mice, which revealed the lowest number of bacteria per area and per host cell for mice treated with cocktail_2_TAT compared to the other treatments (Fig. 8e and f and S3c and d). In line with these results, the abscesses of mice treated with cocktail_2_TAT had the smallest volumes, with an average of 60.6 mm^3^ compared to the control, with an average of 132.7 mm^3^ (Fig. 8d). As previously observed, mice without PGH treatment had lost approximately 10% of their bodyweight after 96 h, whereas mice treated with cocktail_2_TAT had lost significantly less weight (Fig. 8c). Taken together, these *in vivo* results corroborate our *in vitro* findings, demonstrating that both CPPs and synergistic antibacterial effects contribute to the effective killing of (intracellular) S. aureus by PGHs.

10.1128/mBio.00209-20.3 FIG S3 Analysis of S. aureus numbers in pus and tissue of murine abscesses. Animals were treated with 100 μg of LST (green squares), LST_TAT (dark-green triangles), SEP (orange inverted triangles), SEP_TAT (dark-orange diamonds), cocktail_2 (teal circles), and cocktail_2_TAT (navy-blue squares) on days 1, 2, and 3 after infection. All animals including control mice (black circles) received 1 mg flucloxacillin (F) at days 2 and 3 after infection. (a and b) Numbers of viable S. aureus after the different treatments were determined in the pus (a) and tissue (b). The pound symbol (#) indicates a statistically significant difference between cocktail_2_TAT and all other treatments (*P* ≤ 0.0001 for panel a and *P* ≤ 0.05 for panel b). At least seven representative CLSM images of each of the four independent pus smears per treatment group were evaluated for total S. aureus numbers, eukaryotic cell numbers, and intracellular S. aureus. (c and d) Analyses are shown for total bacteria per square millimeter and intracellular bacteria per host cell. Asterisks (*) indicate levels of significance (*, *P* ≤ 0.05; **, *P* ≤ 0.01). Download FIG S3, PDF file, 0.3 MB. Copyright © 2020 Röhrig et al. 2020 Röhrig et al. This content is distributed under the terms of the Creative Commons Attribution 4.0 International license.

## DISCUSSION

In this study, we demonstrated that PGHs selected for activity under relevant conditions and fused to CPPs are promising antibacterial agents that effectively eliminate intracellular S. aureus. The different, highly complex environments found inside and outside cellular compartments pose diverse challenges to PGH activity, as they vary in multiple parameters such as pH, ionic strength, and osmolality (36–38). To accommodate these challenges, we screened PGHs for high activity in multiple buffer systems that simulate conditions found in different intracellular niches of S. aureus, such as the cytoplasm or phagolysosomes. While we have previously described a screening approach to identify PGHs with high activity under the desired conditions (20), selection for PGHs with activity in intracellular compartments has thus far not been pursued. This could explain why previous studies by us and other groups that describe intracellular transduction of PGHs via fusion to CPPs only observed moderate intracellular killing efficacy in cell culture infection models (<1-log reduction) (18, 21). After the systematic selection of PGHs performed here, their CPP-fused derivatives displayed effective intracellular killing, causing reductions in bacterial numbers by several log units.

The selection of the most suitable PGHs was based on a detailed and iterative characterization under four different conditions and in three different activity assays. For this purpose, we developed a dynamic scoring system, which may be further extended to include additional data from other PGHs, activity assays, or buffers. This system also provides valuable information on the performance of individual constructs under the different conditions. For instance, the unique tolerance of SEP for low pH that was observed during the *in vitro* analysis may have contributed to its high efficacy in the murine abscess model. It may also explain why cocktail_2_TAT (containing SEP) was more effective in this model than was cocktail_1_TAT, which instead contained GH15, an enzyme much less active in acidic environments (Fig. 1 and S1). This notion again substantiates the value of a systematic screening strategy preceding further analysis of promising candidates. It should be mentioned that our approach profited from the availability of a comprehensive library of parental and engineered staphylococcal PGHs in our laboratory, which has been established over the course of more than a decade. Efficient screening strategies for laboratories without such preexisting collections, which are instead based on combinatorial approaches, have already been reported and include combining PGH domains on the DNA level (19, 39) or on the protein level (40). Our approach and similar screening approaches may serve to find new lead compounds against intracellular MRSA but might also be transferred to PGHs of other pathogens, such as Streptococcus pyogenes, Listeria monocytogenes, and Bacillus anthracis, which can occur intracellularly (12). Furthermore, such strategies may be used to identify suitable PGHs for applications in various different niches, for example, treatment of bloodstream infections (41).

Despite significantly enhanced intracellular uptake of CPP-fused PGHs compared to their parental counterparts in our study (Fig. 3), this increased internalization did not always translate into more effective killing of intracellular bacteria in the cell culture models (Fig. 5 and Table S1). This may in part be explained by detrimental effects on PGH lytic activity upon fusion to CPPs, as demonstrated for some of the fusion proteins in TKAs (Fig. 2). PGHs have evolved to effectively lyse bacterial cells, and any alterations to their natural structures by molecular engineering may have an impact on their activity, with the severity depending on the individual enzyme and the type of modification (18, 40). Reasons for this might be steric hindrance during CBD binding or PG cleavage, changes in local charge distribution, or altered tertiary structures. This implies that there is often a trade-off between loss in PGH activity and the desired characteristic achieved through protein engineering, in our case, increased uptake. To optimize this trade-off, we aimed at selecting CPPs that had the least impact on PGH activity. TAT, which proved most suitable in this respect, has been well characterized and belongs to the group of cationic, arginine-rich CPPs (42). The fusion of TAT to the C termini of our PGHs, in proximity to the similarly positively charged CBDs, may explain why it had the least impact on activity. In contrast, Pvec, which belongs to the class of secondary amphipathic CPPs (43), had the most detrimental effect on PGH activity. The true potential of our CPP-fused PGH constructs became evident in the murine abscess model, where cocktail_2_TAT was significantly more effective than was cocktail_2, and a similar effect was observed for SEP_TAT and SEP, respectively (Fig. 8). In addition to cellular uptake, CPPs may also facilitate deeper tissue penetration, particularly since bidirectional transport of cargo across eukaryotic cell membranes by some CPPs has been described, thereby possibly enabling cell-to-cell transfer (44, 45). This is supported by our *in vivo* data, where the PGHs were injected in the tissue surrounding the abscess and still had a marked effect on intracellular bacteria within the pus-filled lumen of the abscess.

The observation that cocktail_2_TAT was also significantly more effective than were its individual components suggests that, besides CPP-mediated uptake, synergism also contributed to the potency of this enzyme mixture. Synergistic antibacterial effects when combining PGHs with different PG cleavage specificities have been reported previously (20) and may be explained by the increased accessibility of one PG cleavage site after cleavage of another (46). Here, we demonstrated that synergy can also be observed within intracellular compartments, following CPP-driven PGH uptake. In analogy to the *in vitro* minimal bactericidal concentration (MBC) (47), effective intracellular killing likely depends on reaching a critical PGH concentration within the eukaryotic cell, which is lower for synergistic PGH mixtures than for individual enzymes (20). Another benefit of using mixtures of PGHs that attack multiple PG target sites simultaneously as opposed to individual single-EAD enzymes is that this strategy likely further reduces the chance of resistance development. This is of particular interest when LST is part of the cocktail. As opposed to phage endolysins, various possible mechanisms provoking LST resistance in S. aureus have been described. These include shortening of, or amino acid substitutions, within the relatively weakly conserved pentaglycine bridge, which is the target for the LST M23 endopeptidase (48, 49).

The marked difference in efficacy between cocktail_2_TAT and cocktail_1_TAT in the murine abscess model suggests that their antibacterial activities do not exclusively depend on LST_TAT (which is present in the two cocktails at identical concentrations), but that the non-LST components within these mixtures (i.e., SEP_TAT and GH15_TAT, respectively) have an important impact on the potency of the cocktails. This is further supported by the relatively weak effects of LST and LST_TAT when applied individually *in vivo* (Fig. 8), an observation that seems contradictory to the superior performances of these enzymes in the cell culture experiments (Table S1). One reason for this may lie in the acidic conditions encountered in inflamed tissue and abscesses, which are detrimental for LST activity (50). Our cell culture experiments also revealed that LST (and other PGHs we tested) had remarkable activity against intracellular S. aureus even in the absence of CPPs, which matches previous reports of reductions in intracellular S. aureus numbers after prolonged LST treatment (51). This effect was likely not due to membrane damage, since both parental and CPP-fused PGHs proved to be noncytotoxic even after 15 h of exposure (a finding that is in line with observations by other researchers who tested PGHs on mammalian cell lines and did not find cytotoxicity [52–54]). Instead, it argues for considerable intrinsic internalization capabilities of these enzymes, as has been reported for PlyC (22).

S. aureus has the striking ability to acquire new resistances and develop strategies to survive under stress conditions. The importance of intracellular S. aureus for recurrent infections is evident (55), and consequently, the demand for novel, potent intracellular antimicrobials is high (56). In this context, it is worth pointing out that S. aureus Cowan, which we used *in vitro* and *in vivo* in this study, has been shown to be highly invasive and to form SCVs after exposure to low pH, for example, in phagolysosomes (where it primarily resides) and/or in abscesses (7). This may in part explain the difficulty to treat such infections with conventional antibiotics, which is in line with the observed ineffectiveness of the flucloxacillin treatment used as a control in our animal study. Towards this end, direct fusion of a CPP to the drug vancomycin has been shown to lead to increased intracellular killing *in vitro* and improved pharmacokinetics *in vivo* (57). Furthermore, a cleavable antibody-antibiotic conjugate has been developed for directed targeting of intracellular S. aureus (58). While the results of these studies are highly encouraging, they still rely on classical antibiotics for bacterial inactivation. Therefore, they contribute little to combat the aggravating antibiotic resistance crisis and the associated problem of drug-tolerant and persistent S. aureus (59). PGHs, on the other hand, and phage endolysins in particular, offer the unique advantage of an active and rapid lytic mechanism, with high efficacy against antibiotic-resistant bacteria, metabolically inactive persisters, and biofilms (9, 60). For these reasons, CPP-fused PGHs hold promise as novel and effective future protein therapeutics.

## MATERIALS AND METHODS

### Bacterial strains and culture conditions.

All bacterial strains used in this study are indicated in Table S2. All S. aureus strains were grown from a single colony in tryptic soy broth (TSB) at 37°C. For the GFP-expressing S. aureus RN9623 (61), media were additionally supplemented with 10 μg/ml erythromycin. All E. coli strains used for protein expression were grown at 37°C in Luria-Bertani (LB) medium (10 g/liter tryptone, 5 g/liter yeast extract, 8 g/liter NaCl [pH 7.4]) and on LB agar (LB medium plus 14 g/liter agar) supplemented with suitable antibiotics where necessary (100 μg/ml ampicillin, 30 μg/ml tetracycline, 50 μg/ml kanamycin).

10.1128/mBio.00209-20.5 TABLE S2 Bacterial strains used in the present study. Download Table S2, PDF file, 0.2 MB. Copyright © 2020 Röhrig et al. 2020 Röhrig et al. This content is distributed under the terms of the Creative Commons Attribution 4.0 International license.

### DNA techniques and cloning procedures.

The plasmid constructs used in this work were generated using standard molecular cloning techniques (62). DNA fragments of LST and LysK were amplified from previously published constructs (36) with the Phusion high-fidelity DNA polymerase (New England BioLabs, Allschwil, Switzerland), using the primers listed in Table S3. For CHAPGH15_SH3bALE1, CHAPSEP_SH3b2638a, CHAPTw_SH3b2638a, LysK_LST (previously published as LysK_Lyso [63]), and CHAPK_AmiK_SH3bLST, DNA fragments were purchased from GeneArt (Thermo Fisher Scientific, Waltham, MA, USA). Additionally, fragments encoding fusions of mCherry (64) to a variety of C-terminal CPPs (Table 1) were designed to contain an SacI restriction site between mCherry- and the CPP-coding sequences and purchased from GeneArt. All DNA fragments were inserted between the NdeI and BamHI restriction sites of the pET302/NT-His vector (Invitrogen, Carlsbad, CA, USA), thereby removing the His tag and creating plasmids encoding parental PGHs or mCherry_CPP intermediate constructs. PGH-encoding DNA fragments for the creation of PGH-CPP fusion proteins were amplified from plasmids bearing the parental PGH constructs using primers introducing NdeI and SacI restriction sites. DNA fragments were then inserted between the NdeI and SacI restriction sites of the mCherry_CPP intermediate vectors, thereby replacing the mCherry sequence. All used restriction enzymes were purchased from New England BioLabs. After ligation with T4 DNA ligase (Thermo Fisher Scientific, Waltham, MA, USA), plasmids were transformed into E. coli BL21-Gold(DE3), and their construct identity was confirmed by commercial Sanger sequencing (GATC Biotech AG, Constance, Germany). A list of the plasmids used and created in this study is presented in Table S4.

10.1128/mBio.00209-20.6 TABLE S3 PCR primers used in the present study. Download Table S3, PDF file, 0.2 MB. Copyright © 2020 Röhrig et al. 2020 Röhrig et al. This content is distributed under the terms of the Creative Commons Attribution 4.0 International license.

10.1128/mBio.00209-20.7 TABLE S4 Plasmids used and created in the present study. Download Table S4, PDF file, 0.2 MB. Copyright © 2020 Röhrig et al. 2020 Röhrig et al. This content is distributed under the terms of the Creative Commons Attribution 4.0 International license.

### Microtiter plate-based screening of PGH library for enzymes with activity under intracellular conditions.

We screened 322 PGH constructs from our library using a previously described microtiter plate-based approach (20) in an effort to identify enzymes that retain high staphylolytic activity under conditions encountered within eukaryotic cells. Inoculation, expression, and harvesting of E. coli strains carrying the constructs of interest were performed in 96-well plates, as previously described (20). Each PGH was expressed in three different positions on the same plate. After one freeze-thaw cycle, the 96-well plate bearing the cell pellets was inverted and placed on a metal rack approximately 1 cm above a glass container containing chloroform for 2 × 10 min to expose the bacteria to chloroform vapor, thereby liberating cytosolic proteins and inactivating the E. coli cells. The plate was rotated 180° after 10 min to achieve equal chloroform exposure. S. aureus Newman cells were spiked into sterile buffers and media simulating different extra- and intracellular environments at a concentration of 10^5^ CFU/ml. These included Dulbecco’s phosphate-buffered saline (DPBS) without calcium and magnesium (Thermo Fisher Scientific, Waltham, MA, USA), Dulbecco’s modified Eagle’s medium (DMEM; Thermo Fisher Scientific), intracellular buffer (130 mM KCl, 100 mM NaCl, 20 mM HK_2_PO_4_, 200 mM HEPES, 50 mM succinic acid, 10 mM malic acid, 10 mM pyruvate, 10 mM MgCl_2_ [pH 7] adjusted with KOH) (65) supplemented with 2% albumin fraction V (Carl Roth, Karsruhe, Germany), and lysosomal buffer (140 mM NaCl, 5 mM KCl, 1 mM MgSO_4_, 1 mM CaCl_2_, 1 mM NaH_2_PO_4_, 5 mM d-glucose, 24 mM citric acid, 54 mM Na_2_HPO_4_ [pH 4.7]) supplemented with 1% albumin fraction V (Carl Roth, Karlsruhe, Germany). Lysosomal buffer was produced by adjusting the pH of extracellular buffer (66) to 4.7 using 0.2 M Na_2_HPO_4_ and 0.1 M citric acid at a 24:27 volume ratio. Two hundred microliters of each inoculated buffer or medium was added to each well of the 96-well plate and incubated for 2 h at 37°C under agitation. The mixture was diluted 1:10 in PBS, and 7.5 μl was spotted on a TSB agar plate and incubated at 37°C overnight. Emerging S. aureus colonies within the spots were enumerated, and each PGH construct was scored for its ability to reduce the number of CFU in a given buffer/medium compared to a negative control (E. coli strain expressing no enzyme). A score between 0 and 3 was assigned for each spot, and the scores at each of the three positions on the plate and of each buffer/medium were summed up to obtain a microtiter plate score for each PGH, as follows:

$$\text{Microtiter plate score }\left(\mathrm{PGH}\right)=\overset{\;}{\underset{m}{\sum }}\underset{p}{\sum }(\mathrm{Scor}e_{\mathrm{PGH}}[p,m])$$

where *m* is the medium or buffer, *p* is the position, and *Score_PGH_* is {0, 1, 2, 3}, with 3 for complete clearance (0 CFU), 2 for 1 to 15 CFU, 1 for >15 CFU with visible single colonies, and 0 for >15 CFU without visible single colonies or bacterial lawns.

The 36 PGHs with the highest activity across all four buffers/media were selected for further characterization.

### Protein expression and purification.

Recombinant proteins were expressed in E. coli and purified essentially as previously described (20). In brief, cultures were grown to an optical density at 600 nm (OD_600_) of 0.5 at 37°C under agitation in LB medium modified for protein expression (LB-PE; 15 g/liter tryptone, 8 g/liter yeast extract, 5 g/liter NaCl [pH 7.8]) (67) and cooled on ice. Protein expression was induced with 0.5 mM isopropyl-β-d-thiogalactopyranoside (IPTG) and continued for 18 h at 19°C under agitation. Cells were harvested by centrifugation, and pellets were frozen at −80°C.

For constructs containing His tags, E. coli was resuspended in lysis buffer (50 mM NaH_2_PO_4_, 300 mM NaCl, 10 mM imidazole, 30% glycerol [pH 8]) and lysed in a Stansted pressure cell homogenizer (SPCH-10-230V; Stansted Fluid Power, Harlow, United Kingdom) at 100 MPa. The lysate was cleared of cellular debris by centrifugation and incubated with low-density nickel resin (ABT, Madrid, Spain) in a gravity flow column. After washing, proteins were eluted with elution buffer (50 mM NaH_2_PO_4_, 300 mM NaCl, 250 mM imidazole, 30% glycerol [pH 8]), and fractions containing protein were identified using a NanoDrop ND-1000 spectrophotometer (NanoDrop Technologies, Wilmington, DE). Pooled fractions were dialyzed in Spectra/Por 1 dialysis tubing, with a 6- to 8-kDa molecular weight cutoff (MWCO; Spectrum Laboratories, Rancho Dominguez, CA, USA) against two changes of dialysis buffer (50 mM NaH_2_PO_4_, 300 mM NaCl, 30% glycerol [pH 7.5]) and consequently sterile filtered (0.2-μm pore).

For constructs without a His tag, buffer A (20 mM NaH_2_PO_4_, 20% glycerol [pH 7.4]) was used instead of lysis buffer. After clearance from the cellular debris, the lysate was sterile filtered and loaded onto a HiTrap Sepharose (SP) fast-flow (FF) cation exchange column (GE Healthcare, Uppsala, Sweden) in an Äkta fast-performance liquid chromatography (FPLC) device (GE Healthcare). After washing with 3 column volumes of buffer A, a gradient of 1%/min of buffer B (1 M NaCl, 20 mM NaH_2_PO_4_, 20% glycerol [pH 7.4]) was used to elute the protein. Fractions of 0.5 ml were collected, and those containing protein were dialyzed as described above.

To test for protein identity and purity, 4 μg of protein was analyzed by SDS-PAGE using Mini-Protean TGX-stain-free precast gels (Bio-Rad, Hercules, CA, USA). Impure preparations underwent size exclusion chromatography in a Superdex 200 10/300 GL column (GE Healthcare, Uppsala, Sweden) using gel filtration buffer (50 mM NaH_2_PO_4_, NaCl 500 mM, 10% glycerol [pH 7.4]). All purified proteins were stored on ice at 4°C.

### Antimicrobial activity assays.

As previously reported, the antimicrobial activity of PGHs should ideally be measured by more than one activity assay, since results from different assays can vary on a quantitative level, and each assay may be biased toward different enzyme properties (68). We chose three different *in vitro* activity assays to quantitatively compare the activity of PGH candidates that had been identified by the microtiter plate-based screening through TKAs, TRAs, and SLAs (reviewed in references 8 and 12). TKAs and TRAs were performed in intracellular buffer, DMEM, lysosomal buffer, and PBS. All experiments were performed at least in biological and technical triplicates. Activities in each buffer or medium were translated into scores, as described below. To select for PGHs with robust activity and with a particular focus on intracellular activity, scores in the different formulations were weighted with 4/10, 3/10, 2/10, and 1/10, respectively. To compare PGHs within a given assay, the score of each PGH was normalized to the best-performing PGH within this assay. Scores for all assays were added, with a weighting of 1/6 for SLA, 2/6 for TRA, and 3/6 for the TKA to obtain a relative *in vitro* score for each PGH. The following formula was used for calculation of the relative *in vitro* scores:

$$\text{Relative }\mathrm{in}\;\mathrm{vitro}\text{ score }\left(\mathrm{PGH}\right)=\overset{\;}{\underset{a}{\sum }}\{w_{a}\times \frac{\sum_{b}[{\mathrm{Score}}_{PGH}{\left(a\right)}_{b}\times w_{b}]}{{\text{max}}_{\mathrm{PGH}}\{\sum_{b}[{\mathrm{Score}}_{PGH}{\left(a\right)}_{b}\times w_{b}]\}}\}$$

where *a* is assay; *w_a_* is assay weighting, with 3/6 for TKA, 2/6 for TRA, and 1/6 for SLA; *b* is buffer; *w_b_* is buffer weighting, with 4/10 for intracellular buffer, 3/10 for DMEM, 2/10 for lysosomal buffer, and 1/10 for PBS; and max*_PGH_* is the highest score of all tested PGHs in all buffers.

TKAs were performed essentially as previously described (20). The four different buffers were spiked with 10^6^ CFU/ml of S. aureus Newman. Equimolar amounts of PGHs were mixed with the spiked buffers, resulting in final PGH concentrations of 25 and 100 nM, and the mixtures were stored at 37°C without agitation. After 0, 10, 60, and 180 min, samples were taken, diluted, plated on TSB agar plates, and incubated overnight at 37°C. Bacterial concentrations were determined the following day. To compare different PGHs, scores were assigned based on the observed log reduction. If no bacteria were detected after 10, 60, or 180 min, scores of 20, 10, and 5 were assigned, respectively. If bacteria were still detected after 180 min, reductions of 5, 4, 3, and 2 log units compared to the negative control (no enzyme) at this time point were scored with 4, 3, 2, and 1, respectively. The scores obtained for both tested enzyme concentrations were added and further processed as described above.

TRAs were performed essentially as previously described (36). In brief, we prepared a 2-fold dilution series of each PGH ranging from 200 nM to 12.5 nM in four different buffers. Bacterial suspensions were prepared from frozen S. aureus Newman substrate cells in each buffer, and 100 μl of the bacterial suspensions was mixed with 100 μl of each dilution of the PGHs in a 96-well plate, so that the initial OD_600_ of the suspension was 1. The decrease in optical density over time was monitored for 1 h at 30-s intervals with a FLUOstar Omega plate reader (BMG Labtech, Ortenberg, Germany). The resulting lysis curves were corrected for the negative controls (no enzyme) and fitted to a 5-parametric sigmoidal function, as described before (69), using SigmaPlot 13 (Systat Software, San Jose, CA). The specific activity of each PGH, expressed as the ΔOD_600_ min^−1 ^μM^−1^, was determined within the linear activity range of the enzyme, as previously described (70). Specific activity values served as scores for the TRA and were further processed as described above.

For SLAs, serial dilutions of PGHs were spotted onto freshly plated S. aureus Newman lawns on square TSB agar plates, as previously described (71). A log-phase culture of S. aureus Newman was diluted 1:10, and 20 ml was used to cover the agar plate. After decanting the residual liquid, plates were dried for 10 min, and 10 μl of purified PGHs at concentrations of 10 μM, 1 μM, and 0.1 μM was spotted on the plate. PBS served as a negative control. The plates were incubated at 37°C overnight. Enzymes that produced a cleared lysis zone in the S. aureus lawn at concentrations of 0.1, 1, and 10 μM were rated with scores of 3, 2, and 1, respectively. Scores from multiple replicates were averaged and processed as described above.

### Eukaryotic cell culture.

A549 (ATCC CCL-185) adenocarcinomic human alveolar basal epithelial cells (A549 cells) and 3T3-L1 (ATCC CL-173) primary murine fibroblast-derived adipocytes (3T3-L1 cells) were grown in Dulbecco’s modified Eagle medium (DMEM [Gibco] plus 4.5 g/liter d-glucose and pyruvate) with 10% fetal bovine serum (FBS; Thermo Fisher Scientific, Waltham, MA, USA) (72, 73). MG-63 (ATCC CRL-1427) osteosarcoma cells (MG-63 cells) were grown in minimum essential medium (MEM [Gibco] plus Earl’s salts and l-glutamine) with 10% FBS (74). Cells were incubated at 37°C and 5% CO_2_ and maintained at ≤90% confluence for A549 and MG-63 cells and ≤80% confluence for 3T3-L1 cells. Differentiation of 3T3-L1 cells was performed as described before (75), with the following adjustments. The day after seeding, differentiation was induced by adding medium supplemented with 0.5 mM 3-isobutyl-1-methylxanthine (IBMX; Sigma-Aldrich, St. Louis, MO, USA), 1 μg/ml insulin (Sigma-Aldrich), 1 μM rosiglitazone (Sigma-Aldrich), and 1 μM dexamethasone (Sigma-Aldrich). Two days after induction, the medium was changed to contain only 1 μg/ml insulin. Four days after induction, normal DMEM was used again, and the presence of lipid droplets was confirmed by light microscopy. The maximal passage numbers used were 23, 21, and 10 for A549, MG-63, and 3T3-L1 cells, respectively.

### Europium labeling and quantification of PGH uptake.

Labeling of PGHs and PGH-CPP fusion proteins with the fluorescent {{2,2′,2″,2‴-{4′-{[(4,6-dichloro-1,3,5-triazin-2-yl)amino]biphenyl-4-yl}-2,2′:6′,2″-terpyridine-6,6′′-diyl}bis-(methylenenitrilo)}tetrakis(acetato)} europium(III) (DTBTA-Eu^3+^) chelate was performed essentially as described before (76). Briefly, 12 parts of 33.3 mg/ml {2,2′,2″,2‴-{[4′-(aminobiphenyl-4-yl)-2,2′:6′,2″-terpyridine-6,6″-diyl]bis(methylenenitrilo)}-tetrakis(acetato)} europium(III) (ATBTA-Eu^3+^; TCI Chemicals, Tokyo, Japan) in 0.1 M sodium acetate buffer (pH 4.9) were mixed with five parts of 17.2 mg/ml cyanuric chloride in acetone for 30 min at room temperature. The mixture was precipitated in pure acetone in a dropwise manner and washed with acetone two times, followed by vacuum drying for 1 h. Synthesis of the DTBTA-Eu^3+^ molecule was confirmed by electrospray ionization-mass spectrometry (ESI-MS) at the Functional Genomics Center Zurich (FGCZ, Zurich, Switzerland). ESI-MS analysis was performed within a mass range between 50 and 5,000 Da, with a sampling cone energy of 40 V. Proteins were dialyzed into carbonate buffer (300 mM NaCl [pH 9.1]), and DTBTA-Eu^3+^ was mixed with purified proteins at a molar ratio of 4:1 and incubated for 2.5 h under agitation at room temperature. Unbound label was removed by gel filtration chromatography on a Sephadex G-25 gravity flow column (PD-10, GE Healthcare, Uppsala, Sweden). Protein concentrations were determined using the Pierce bicinchoninic acid (BCA) protein assay kit (Thermo Fisher Scientific, Waltham, MA, USA), according to the manufacturer’s instructions, and protein purity was confirmed by SDS-PAGE. Labeled proteins were incubated with A549 cells grown in a 24-well tissue culture plate (Bioswisstec, Schaffhausen, Switzerland) at concentrations of 0.5, 1.0, and 2.0 μM for 4 h. Cells were then washed three times with medium (DMEM or MEM) to remove PGHs. Cell integrity was confirmed by microscopy before and after washing. Eukaryotic cells were detached by adding 60 μl trypsin (trypsin–0.25% EDTA; Gibco) and lysed by adding 240 μl of 0.1% Triton X-100 (Sigma-Aldrich, St. Louis, MO, USA) for 3 min at 37°C. Additionally, the mixture was mashed by repeatedly pipetting up and down. A 2-fold dilution series of each labeled PGH was prepared in the same buffer. Time-resolved fluorescence of the lysate and the dilution series (for generating a standard curve) was measured in a 384-well plate in an Infinite M1000 reader (Tecan, Männedorf, Switzerland). PGH concentrations in the lysate were calculated from the standard curve.

### Microscopy of intracellular S. aureus.

In each well of a 2-well imaging chamber (ibidi GmbH, Martinsried, Germany), 1.25 × 10^5^ MG-63 cells were seeded and cultured for 24 h. The GFP-producing S. aureus strain RN9623 was washed once, resuspended in PBS, and used for infection of cultured cells at a multiplicity of infection (MOI) of 1 for 1 h. At the start of infection, 225 nM death-associated protein kinase (DAPK) inhibitor (Merck, Darmstadt, Germany) and 20 μM (3S)-5-(2,6-difluorophenoxy)-3-[[(2S)-3-methyl-1-oxo-2-[(2-quinolinylcarbonyl)amino]butyl]amino]-4-oxo-pentanoic acid (Q-VD-OPh) (Sigma-Aldrich, St. Louis, MO, USA) were added to minimize the cytotoxic effects of S. aureus. Subsequently, eukaryotic cells were washed with prewarmed medium, and medium supplemented with 1 mg/ml flucloxacillin was added for 1 h to kill all extracellular bacteria (7, 59). The DNA stain Hoechst 33342 (Thermo Fisher Scientific, Waltham, MA, USA) and the cell membrane stain FM4-64 (Thermo Fisher Scientific) were added at final concentrations of 1 μg/ml and 10 μg/ml, respectively. After 20 min, PGHs, PGH-CPP fusion proteins, or PBS was added to the cells at a concentration of 2 μM and incubated for up to 5 h at 37°C and 5% CO_2_. Imaging was started immediately after the addition of PGH and was performed on a Leica TCS SPE confocal system (Leica Microsystems GmbH, Germany) equipped with an HCX PL Fluotar 63× 1.30 oil objective. A z-stack of 20 μm with a z-slice thickness of 0.5 μm was imaged every 5 min, thereby creating time-resolved 3-dimensional reconstructions of the infected cells over a treatment duration of >4 h. Hoechst 33342, GFP, and FM4-64 were excited at λ of 405 nm, 488 nm, and 532 nm, respectively. Image analysis was performed with the Imaris Software (Bitplane AG, Zurich, Switzerland).

### Intracellular killing assay.

In each well of a 24-well tissue culture plate (Bioswisstec, Schaffhausen, Switzerland), 2.5 × 10^5^ A549 or MG-63 cells were seeded 24 h before infection. For the 3T3-L1 cell line, 6.25 × 10^4^ cells were seeded in a 24-well plate 7 days before infection and differentiated as described above. On the day of infection, a log-phase culture of S. aureus Newman, Cowan, or USA300 JE2 was washed once and resuspended in PBS. The MOIs used for the infection of eukaryotic cells were 10, 1, and 1, respectively. The higher MOI for Newman was chosen because of its low propensity to invade eukaryotic cells, which is most likely due to the truncation of fibronectin binding proteins (77). Cells were infected for 3 h at 37°C and 5% CO_2_, washed with prewarmed medium (DMEM or MEM), and incubated with medium supplemented with 1 mg/ml flucloxacillin, which was determined to be at least 40× higher than the MIC of each strain. After 1 h, PGHs or PGH-CPP fusion proteins were added to the cells at 2 μM and incubated for 4 or 15 h at 37°C and 5% CO_2_. Controls without S. aureus infection and without PGH treatment were included in the assay. Additionally, the extracellular medium was plated to confirm the absence of extracellular S. aureus. Cells were washed three times with medium (DMEM or MEM) to remove flucloxacillin and enzymes. Cell integrity was confirmed by microscopy, and eukaryotic cells were detached by adding 60 μl trypsin (trypsin–0.25% EDTA; Gibco) and lysed by adding 240 μl of 0.1% Triton X-100 (Sigma-Aldrich, St. Louis, MO, USA) for 3 min at 37°C. Additionally, the mixture was mashed by repeated pipetting, and 700 μl of cold PBS was subsequently added to the mixture. The cell lysate was serially diluted in PBS, plated on tryptic soy agar (TSA) plates, and incubated at 37°C overnight. On the following day, emerging colonies were enumerated.

### Intracellular synergy assays.

To determine the synergistic effects of mixtures of PGH-CPP fusion proteins inside eukaryotic cells, intracellular killing assays were performed in MG-63 cells infected with S. aureus USA300 JE2 as described above, with the following modifications. In a preliminary dose-response experiment, 2-fold serial dilutions ranging from 0.04 to 0.0025 μM for LST_TAT and from 4 to 0.25 μM for CHAPGH15_SH3bALE1_TAT, LysK_TAT, and CHAPSEP_SH3b2638a_TAT were added to the eukaryotic cells for 15 h. For all enzymes, concentrations yielding approximately the same log reduction in intracellular S. aureus numbers were determined. To test for synergy between LST_TAT and any of the other three PGHs, log reductions caused by two individual enzymes in the intracellular killing assay at these predetermined concentrations were compared with those caused by a mixture of the two proteins at half these concentrations.

### Cytotoxicity assay.

Cytotoxicity assays were performed according to the manufacturer’s instructions of the Pierce LDH cytotoxicity assay kit (Thermo Fisher Scientific, Waltham, MA, USA). Briefly, the supernatant of A549 cells was tested for its LDH content either with or without infection by S. aureus USA300 JE2. In both cases, cells were treated with 2 μM PGHs for 15 h, as described for the intracellular killing assay. The absorption of the supernatant at 490 nm (signal) and 680 nm (background) was measured with a FLUOstar Omega plate reader (BMG Labtech, Ortenberg, Germany). After background subtraction, cytotoxicity was determined by division of the signal of the samples by the signal of a maximum LDH activity obtained through lysis of all cells by a detergent and subsequent multiplication by 100%.

### Murine abscess model.

The Institutional Animal Care and Use Committee of the University of Zurich approved the study under protocol ZH050/18, and all animal experiments conducted in this study were approved by the Cantonal Veterinary Office Zurich.

S. aureus Cowan was grown to logarithmic phase, and 10^8^ CFU were mixed 1:1 with Cytodex beads (Sigma-Aldrich, St. Louis, MO, USA) and injected subcutaneously into the flanks of 7- to 8-week-old female C57BL/6 mice (Janvier Laboratory, France), as previously described (35). PBS or either 4, 20, or 100 μg of PGHs was injected daily subcutaneously close to the abscess area over 3 days starting at 24 h postinfection. All PGH cocktail formulations were equimolar mixtures of the two proteins of interest. Additionally, all mice received 1 mg flucloxacillin (Actavis, Parsippany-Troy Hills Township, NJ, USA) intraperitoneally at days 2 and 3 to kill extracellular S. aureus. The weight of each animal was monitored daily. The mice were sacrificed 4 days postinfection. Abscess dimensions were measured, and abscesses were excised. Pus and the surrounding tissue were homogenized, serially diluted, and plated for bacterial enumeration. In order to quantify intracellular bacteria, harvested pus was additionally incubated with 1 mg/ml flucloxacillin for 2 h *ex vivo* to kill extracellular bacteria, washed, lysed with water, and plated for bacterial enumeration. The abscess area and volume were calculated as previously described (78).

In addition, abscess pus was stained with 10 μg/ml FM 4-64FX (Thermo Fisher Scientific, Waltham, MA, USA) and 1 μg/ml Hoechst 33342 (Thermo Fisher Scientific) before fixation with 4% paraformaldehyde (Sigma-Aldrich, St. Louis, MO, USA). PBS with 0.1% albumin fraction V (Carl Roth, Karsruhe, Germany), 2% FBS (Thermo Fisher Scientific), and 1% saponin (Sigma-Aldrich) was used to block and permeabilize the samples. S. aureus was labeled with mouse anti-S. aureus primary antibody Ab37644 (Abcam, Cambridge, UK) and with Alexa Fluor 488-conjugated goat anti-mouse secondary antibody Ab150113 (Abcam). The samples were visualized as described above.

### Statistical analysis.

All experiments were performed at least in biological triplicates, and statistical analysis was performed in Prism v8.0 (GraphPad Software, San Diego, CA, USA). The CFU counts of all experiments were log transformed prior to analysis. A two-way analysis of variance (ANOVA) with Tukey’s correction for multiple comparisons was used to analyze the difference in (i) the uptake of europium-labeled PGHs with or without CPPs, (ii) intracellular CFU after exposure to different PGHs with and without CPPs, and (iii) the difference in intracellular CFU after exposure to these agents for different periods (4 h and 15 h). Differences in intracellular CFU in synergy experiments were analyzed using a one-way ANOVA with Tukey’s correction for multiple comparisons. For *in vivo* experiments, the weight loss data of mice were analyzed by a two-way ANOVA with Dunnett’s correction for multiple comparisons. Differences in CFU counts and abscess size were analyzed using a one-way ANOVA with Tukey’s correction for multiple comparisons.

### Data availability.

The data supporting the findings of this study are available within the article and its supplemental material files, or from the corresponding author on request.

10.1128/mBio.00209-20.9 MOVIE S2 Time-resolved three-dimensional CLSM video of eukaryotic cells infected with S. aureus and treated with LST. The GFP-expressing S. aureus RN9623 (green) was used for infection of MG-63 cells, and flucloxacillin was added to the medium after infection. DNA was stained with Hoechst 33342 (blue) and cell membranes with FM464 (red). Treatment with LST was initiated at *t* = 0 h. To demonstrate intracellular localization of S. aureus, the red channel was imaged at *t* = 0 h but not used afterwards to minimize additional photobleaching. z-stacks were recorded every 5 minutes for 5 h. Scale bar = 30 μm. Download Movie S2, AVI file, 8.2 MB. Copyright © 2020 Röhrig et al. 2020 Röhrig et al. This content is distributed under the terms of the Creative Commons Attribution 4.0 International license.

10.1128/mBio.00209-20.10 MOVIE S3 Time-resolved three-dimensional CLSM video of eukaryotic cells infected with S. aureus and treated with LST_TAT. The GFP-expressing S. aureus RN9623 (green) was used for infection of MG-63 cells, and flucloxacillin was added to the medium after infection. DNA was stained with Hoechst 33342 (blue) and cell membranes with FM464 (red). Treatment with LST_TAT was initiated at *t *= 0 h. To demonstrate intracellular localization of S. aureus, the red channel was imaged at *t *= 0 h but not used afterwards to minimize additional photobleaching. z-stacks were recorded every 5 minutes for 5 h. Scale bar = 30 μm. Download Movie S3, AVI file, 8.2 MB. Copyright © 2020 Röhrig et al. 2020 Röhrig et al. This content is distributed under the terms of the Creative Commons Attribution 4.0 International license.
