# Supplementary material for: Targeting Hidden Pathogens: Cell-Penetrating Enzybiotics Eradicate Intracellular Drug-Resistant Staphylococcus aureus
Source: mBio. 2020 Apr 14;11(2):e00209-20. doi: 10.1128/mBio.00209-20 (PMC7157818; doi:10.1128/mBio.00209-20)
Supplement: FIG S1 [file mBio.00209-20-sf001.pdf]

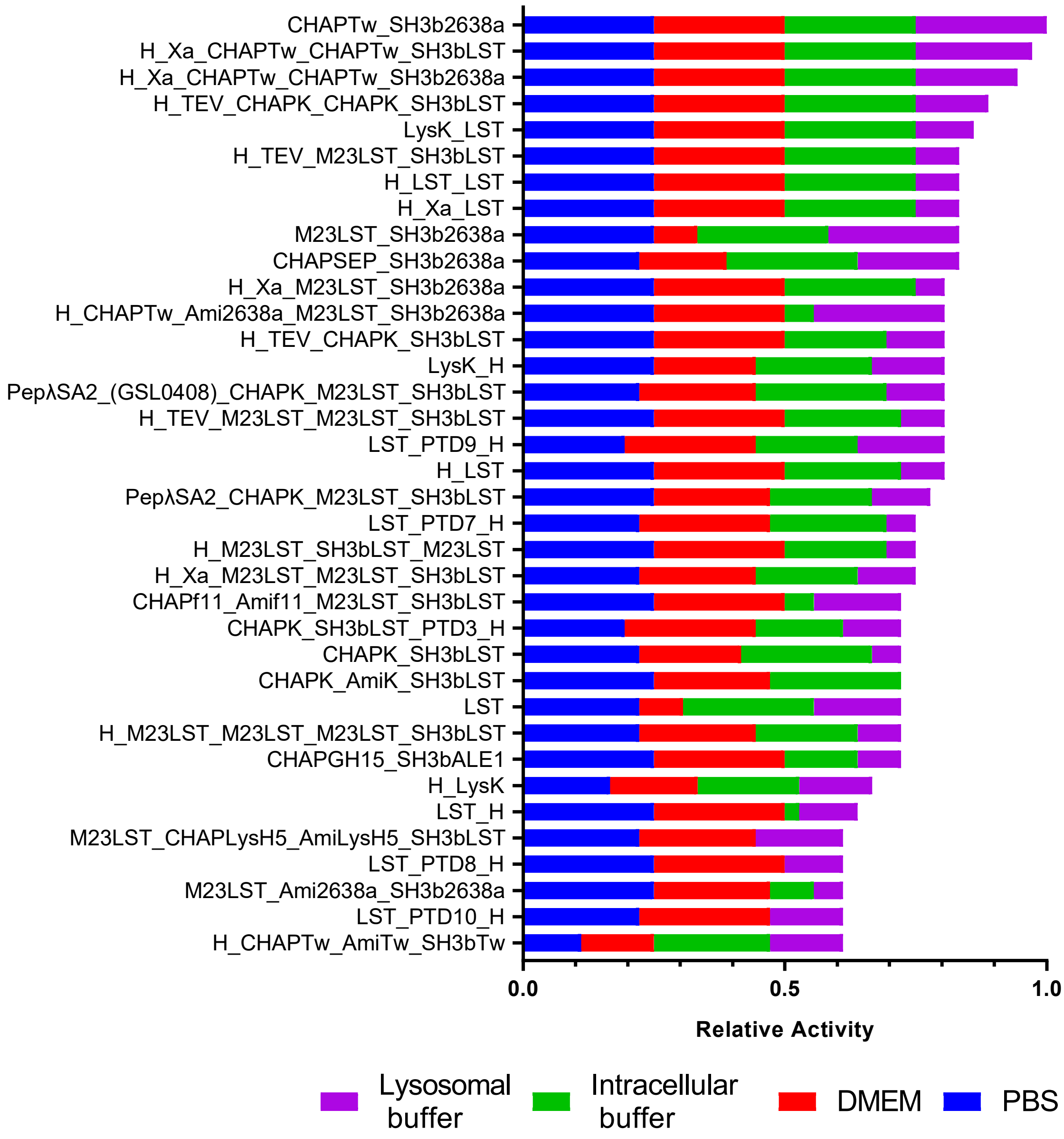

FIG S1 Relative activity scores of the 36 most potent staphylolytic PGHs identified by microtiter plate-based screening simulating extra- and intracellular conditions. Composite scores are the sum of individual scores derived from activities in 4 different buffers or media. Constructs are named according to their domain structure and origin of individual domains. CHAP, CHAP endopeptidase domain; M23, M23 endopeptidase domain; Pep, endolysin-derived endopeptidase domain; Ami, N-acetylmuramoyl-l-alanine amidase domain; SH3b, Src-homology 3b domain; Tw, phage Twort endolysin; 2638a, phage 2638a endolysin; K, phage K endolysin (LysK); SEP, phage SEP1 endolysin; f11, phage phi11 endolysin; GH15, phage GH15 endolysin; LysH5, phage H5 endolysin; ALE1, bacteriocin ALE1; LST, lysostaphin; Xa, factor Xa protease cleavage site; TEV, tobacco etch virus cleavage site; PTD, protein transduction domain; H, His-Tag.
