# Supplementary material for: Targeting Hidden Pathogens: Cell-Penetrating Enzybiotics Eradicate Intracellular Drug-Resistant Staphylococcus aureus
Source: mBio. 2020 Apr 14;11(2):e00209-20. doi: 10.1128/mBio.00209-20 (PMC7157818; doi:10.1128/mBio.00209-20)
Supplement: TABLE S1 [file mBio.00209-20-st001.pdf]

TABLE S1 Killing of intracellular *S. aureus* strains by PGH and PGH-CPP constructs in three eukaryotic cell lines after exposure for 4 h

Cell line	A549			MG-63			3T3-L1		
***S. aureus* strain and MOI**	**Newman** MOI 10	**Cowan** MOI 1	**USA300 JE2** MOI 1	**Newman** MOI 10	**Cowan** MOI 1	**USA300 JE2** MOI 1	**Newman** MOI 10	**Cowan** MOI 1	**USA300 JE2** MOI 1
**Intracellular *S. aureus* in control [log CFU/ml]**	4.00 ± 0.24	5.94 ± 0.16	5.45 ± 0.15	3.65 ± 0.17	5.22 ± 0.13	4.90 ± 0.15	4.83 ± 0.12	5.30 ± 0.19	5.57 ± 0.15
**construct**	**Reduction of intracellular *S. aureus* by PGH/PGH-CPP treatment [log CFU/ml]**								
LST	0.46 ± 0.3 ns	1.6 ± 0.02 **	2.55 ± 0.36 ****	1.21 ± 0.24 ns	1.57 ± 0.61 ns	2.29 ± 0.85 *	1.52 ± 0.23 ****	2.33 ± 0.45 ****	2.46 ± 0.1 ****
LST_TAT	n.d. ***	4.13 ± 0.32 ****	n.d.****	n.d. ns	n.d. ****	n.d. ****	n.d. ****	n.d. ****	n.d. ****
LST_Penetratin	n.d. ***	4.18 ± 0.22 ****	3.01 ± 0.25 ****	n.d. ns	n.d. ****	n.d. ****	n.d. ****	n.d. ****	4.29 ± 0.1 ****
LST_Pvec	n.d. ***	4.2 ± 0.26 ****	1.45 ± 0.14 **	n.d. ns	2.95 ± 0.42 ***	3.13 ± 0.17 ***	3.48 ± 0.1 ****	n.d. ****	4.21 ± 0.13 ****
KLST	1.24 ± 0.41 ns	0.52 ± 0.5 ns	0.79 ± 0.13 ns	n.d. ****	0.65 ± 0.26 ns	2.41 ± 0.25 ****	n.a.		
KLST_Pen	1.42 ± 0.3 ns	1.82 ± 0.19 ns	1.52 ± 0.63 ns	n.d. ****	1.14 ± 0.12 *	2.84 ± 0.13 ****			
LysK	0.46 ± 0.08 ns	-0.06 ± 0.09 ns	0.54 ± 0.2 ns	0.06 ± 0.07 ns	0.1 ± 0.04 ns	0.27 ± 0.08 ns	1.02 ± 0.12 ***	0.4 ± 0.12 ns	1.22 ± 0.05 ****
LysK_TAT	0.71 ± 0.03 ns	0.01 ± 0.07 ns	0.85 ± 0.24 ns	0.38 ± 0.18 ns	0.18 ± 0.05 ns	0.43 ± 0.19 ns	1.16 ± 0.09 ****	0.53 ± 0.14 ns	0.85 ± 0.11 **
LysK_Phy1	0.63 ± 0.07 ns	-0.05 ± 0.06 ns	0.61 ± 0.17 ns	0.48 ± 0.11 ns	0.13 ± 0.05 ns	0.56 ± 0.23 ns	0.96 ± 0.21 ***	0.7 ± 0.13 *	1.28 ± 0.03 ****
LysK_Phy2	0.54 ± 0.19 ns	0.02 ± 0.07 ns	0.62 ± 0.22 ns	0.55 ± 0.2 ns	0.46 ± 0.06 ns	0.74 ± 0.21 ns	1.32 ± 0.29 ****	0.84 ± 0.11 **	1.14 ± 0.11 ****
GH15	0.35 ± 0.19 ns	0.96 ± 0.15 ns	0.19 ± 0.02 ns	0.87 ± 0.37 ns	0.11 ± 0.19 ns	0.87 ± 0.3 ns	0.72 ± 0.08 ns	0.54 ± 0.07 ns	1.06 ± 0.11 **
GH15_TAT	1.16 ± 0.15 ns	1.12 ± 0.05 ns	0.44 ± 0.28 ns	n.d. ns	1.2 ± 0.24 ns	1.78 ± 0.53 ns	1.14 ± 0.24 **	1.13 ± 0.23 **	1.64 ± 0.07 ****
GH15_Penetratin	0.83 ± 0.01 ns	0.88 ± 0.1 ns	0.13 ± 0.26 ns	1.28 ± 0.3 ns	1.06 ± 0.28 ns	1.44 ± 0.73 ns	1.25 ± 0.16 ***	0.75 ± 0.02 ns	1.56 ± 0.11 ****
GH15_Pvec	0.39 ± 0.09 ns	0.81 ± 0.1 ns	0.34 ± 0.22 ns	1.39 ± 0.21 ns	0.64 ± 0.34 ns	0.72 ± 0.8 ns	0.98 ± 0.13 *	0.51 ± 0.27 ns	1.09 ± 0.11 **
SEP	0.57 ± 0.03 ns	0.21 ± 0.23 ns	-0.05 ± 0.25 ns	0.47 ± 0.13 ns	0.48 ± 0.11 ns	-0.06 ± 0.08 ns	0.54 ± 0.04 ns	0.39 ± 0.1 ns	0.56 ± 0.02 ns
SEP _TAT	0.44 ± 0.03 ns	0.22 ± 0.2 ns	-0.51 ± 0.36 ns	0.38 ± 0.21 ns	-0.09 ± 0.31 ns	0.12 ± 0.14 ns	0.46 ± 0.06 ns	0.37 ± 0.09 ns	0.42 ± 0.15 ns
SEP _Pene	0.44 ± 0.01 ns	0.01 ± 0.18 ns	-0.17 ± 0.18 ns	0.52 ± 0.25 ns	0.55 ± 0.08 ns	0.23 ± 0.06 ns	0.56 ± 0.12 ns	0.04 ± 0.18 ns	0.78 ± 0.08 **
SEP _Pvec	0.34 ± 0.07 ns	-0.2 ± 0.02 ns	0.31 ± 0.23 ns	0.45 ± 0.08 ns	0.31 ± 0.08 ns	0.11 ± 0.2 ns	0.34 ± 0.07 ns	0 ± 0.24 ns	0.62 ± 0.03 ns
SEP _Phy1	0.41 ± 0.11 ns	-0.09 ± 0.07 ns	0.15 ± 0.37 ns	0.17 ± 0.16 ns	-0.06 ± 0.05 ns	-0.11 ± 0.13 ns	0.3 ± 0.04 ns	0.24 ± 0.07 ns	0.43 ± 0.06 ns
SEP _Phy2	1.59 ± 1.01 ns	0.17 ± 0.2 ns	0.2 ± 0.24 ns	0.42 ± 0.1 ns	0.17 ± 0.07 ns	-0.29 ± 0.08 ns	0.32 ± 0.12 ns	0.23 ± 0.06 ns	0.39 ± 0.1 ns
CKAK	-0.29 ± 0.29 ns	0.63 ± 0.19 ns	0.27 ± 0.25 ns	0.43 ± 0.19 ns	0.32 ± 0.22 ns	0.7 ± 0.38 ns	0.57 ± 0.23 ns	0.91 ± 0.1 *	1.15 ± 0.14 **
CKAK _Penetratin	n.a.			0.62 ± 0.26 ns	0.32 ± 0.09 ns	0.95 ± 0.42 ns	0.56 ± 0.2 ns	0.97 ± 0.04 *	0.99 ± 0.13 *

n.d., reduction below detectable level; ns, not significant; n.a., data not available; asterisks (*) indicate p-values (* = p≤0.05, ** = p≤0.01, *** = p≤0.001, **** = p≤0.0001); treatments significantly reducing *S. aureus* numbers compared to the control are highlighted in green.
