# Supplementary material for: Targeting Hidden Pathogens: Cell-Penetrating Enzybiotics Eradicate Intracellular Drug-Resistant Staphylococcus aureus
Source: mBio. 2020 Apr 14;11(2):e00209-20. doi: 10.1128/mBio.00209-20 (PMC7157818; doi:10.1128/mBio.00209-20)
Supplement: FIG S2 [file mBio.00209-20-sf002.pdf]

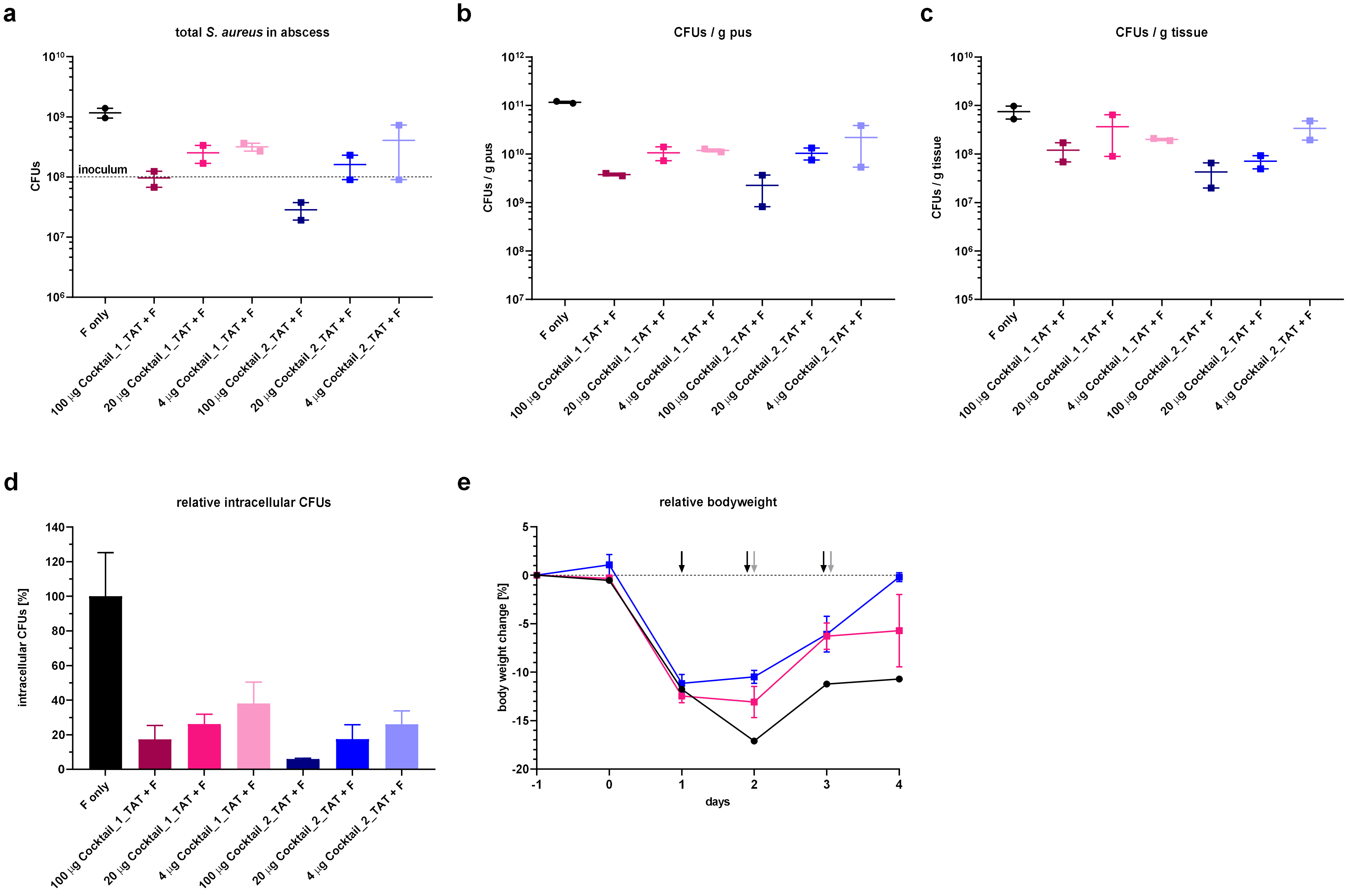
a
total S. aureus in abscess
CFUs
inoculum
F only
100 µg Cocktail_1_TAT + F
20 µg Cocktail_1_TAT + F
4 µg Cocktail_1_TAT + F
100 µg Cocktail_2_TAT + F
20 µg Cocktail_2_TAT + F
4 µg Cocktail_2_TAT + F
b
CFUs / g pus
c
CFUs / g tissue
d
relative intracellular CFUs
intracellular CFUs [%]
e
relative bodyweight
body weight change [%]
days

FIG S2 Efficacy of different doses of Cocktail_1_TAT and Cocktail_2_TAT against *S. aureus* Cowan in a murine abscess model. Cocktail_1_TAT was tested at 4 µg (■), 20 µg (■) and 100 µg (■), and Cocktail_2_TAT was tested at 4 µg (■), 20 µg (■) and 100 µg (■). All animals received 1 mg flucloxacillin (F) at days 2 and 3 post infection. Total *S. aureus* numbers in the abscesses after 4 days (**a**) were determined as the sum of bacterial numbers in pus (**b**) and surrounding tissue (**c**). Relative numbers of intracellular bacteria in pus after treatment of pus with flucloxacillin *ex vivo* (**d**). Relative mean bodyweight of mice treated with Cocktail_1_TAT (■), Cocktail_2_TAT (■), or buffer (●) over the time of infection and treatment (**e**). Error bars show S.E.M. Administration of PGHs and flucloxacillin is indicated by black and grey arrows, respectively.
