# Supplementary material for: Targeting Hidden Pathogens: Cell-Penetrating Enzybiotics Eradicate Intracellular Drug-Resistant Staphylococcus aureus
Source: mBio. 2020 Apr 14;11(2):e00209-20. doi: 10.1128/mBio.00209-20 (PMC7157818; doi:10.1128/mBio.00209-20)
Supplement: FIG S3 [file mBio.00209-20-sf003.pdf]

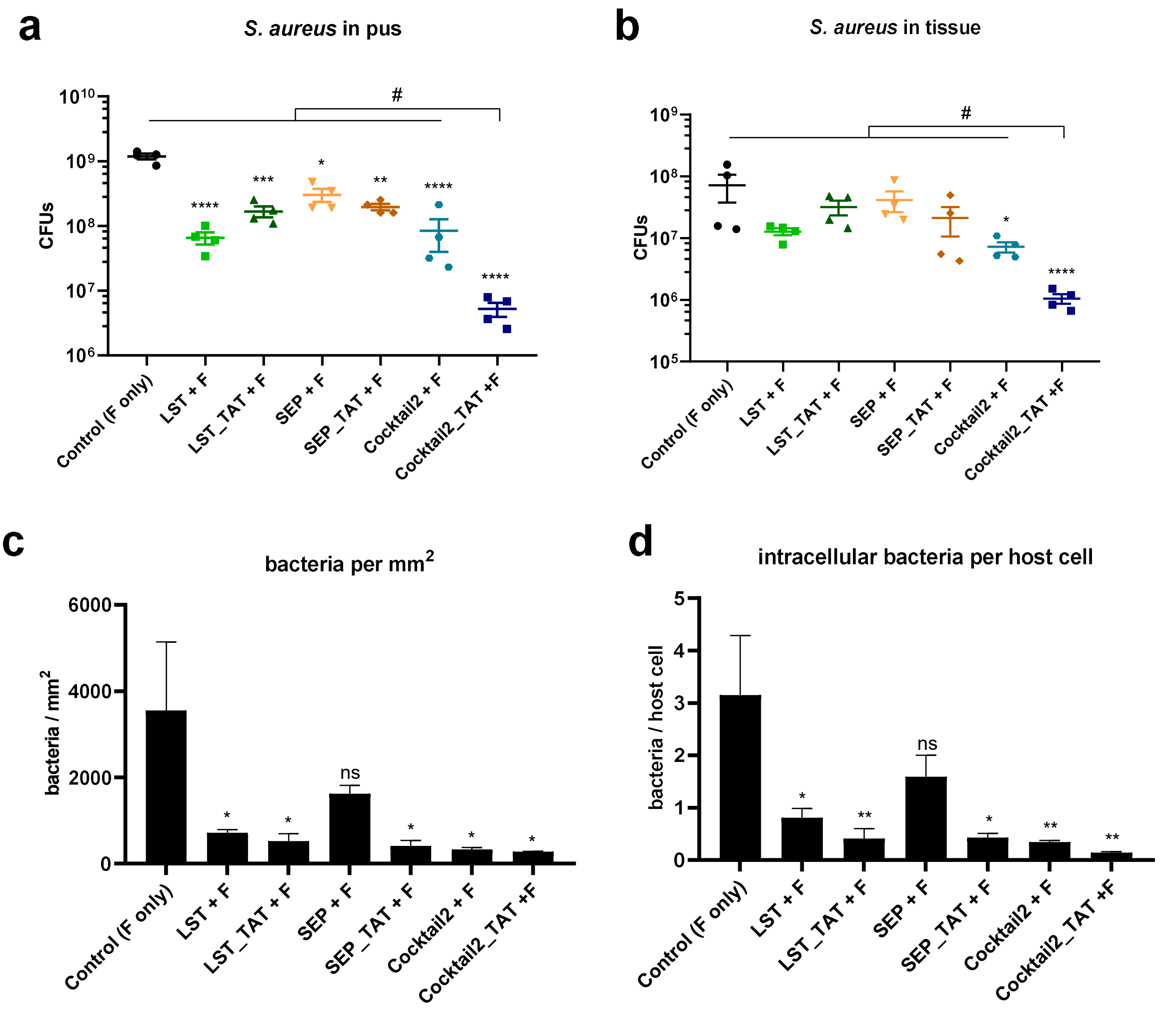

FIG S3 Analysis of *S. aureus* numbers in pus and tissue of murine abscesses. Animals were treated with 100 µg of LST (■), LST_TAT (▲), SEP (▼), SEP_TAT (♦), Cocktail_2 (●) and Cocktail_2_TAT (■) on day 1, 2 and 3 after infection. All animals including control mice (●) received 1 mg flucloxacillin (F) at day 2 and 3 after infection. Numbers of viable *S. aureus* after the different treatments were determined in the pus (**a**) and tissue (**b**). The hash symbol (#) indicates a statistically significant difference between Cocktail_2_TAT and all other treatments ($p \leq 0.0001$ for (a) and $p \leq 0.05$ for (b)). At least seven representative CLSM images of each of the four independent pus smears per treatment group were evaluated for total *S. aureus* numbers, eukaryotic cell numbers, and intracellular *S. aureus*. Analyses are shown for total bacteria per mm^2 (**c**) and intracellular bacteria per host cell (**d**). Asterisks (*) indicate levels of significance (*, $p \leq 0.05$; **, $p \leq 0.01$).
