# Supplementary material for: Targeting Hidden Pathogens: Cell-Penetrating Enzybiotics Eradicate Intracellular Drug-Resistant Staphylococcus aureus
Source: mBio. 2020 Apr 14;11(2):e00209-20. doi: 10.1128/mBio.00209-20 (PMC7157818; doi:10.1128/mBio.00209-20)
Supplement: TABLE S2 [file mBio.00209-20-st002.pdf]

TABLE S2 Bacterial strains used in the present study

Species	Strain	Source	Properties
S. aureus	Newman	ATCC 25904	Clinical isolate (osteomyelitis)
S. aureus	Cowan	ATCC 12598	Clinical isolate (septic arthritis)
S. aureus	USA300 JE2	NR-46543, Network on Antimicrobial Resistance in *Staphylococcus aureus* (NARSA) (Reference 1)	Clinical isolate (skin and soft tissue infection), plasmid cured, Met^R
S. aureus	RN9623	(Reference 2)	RN4220(pCN57), Ery^R
E. coli	BL21 GOLD (DE3)	Agilent Technologies, Santa Clara, USA	Tet^R
E. coli	XL1-BLUE MRF’	Stratagene, San Diego, USA	Tet^R
E. coli	SURE	Stratagene, San Diego, USA	Kan^R
E. coli	ClearColi BL21 GOLD (DE3)	Lucigen, Middleton, Wisconsin, USA	Lipoteichoic acid-deficient mutant (endotoxin free)

Met, methicillin; Ery, erythromycin; Tet, tetracycline; Kan, kanamycin; ^{R}, resistance

References

1. Fey PD, Endres JL, Yajjala VK, Widhelm TJ, Boissy RJ, Bose JL, Bayles KW. 2013. A genetic resource for rapid and comprehensive phenotype screening of nonessential *Staphylococcus aureus* genes. mBio 4:e00537-12.
2. Charpentier E, Anton AI, Barry P, Alfonso B, Fang Y, Novick RP. 2004. Novel cassette-based shuttle vector system for gram-positive bacteria. Appl Environ Microbiol 70:6076-85.
