# Supplementary material for: Targeting Hidden Pathogens: Cell-Penetrating Enzybiotics Eradicate Intracellular Drug-Resistant Staphylococcus aureus
Source: mBio. 2020 Apr 14;11(2):e00209-20. doi: 10.1128/mBio.00209-20 (PMC7157818; doi:10.1128/mBio.00209-20)
Supplement: TABLE S3 [file mBio.00209-20-st003.pdf]

TABLE S3 PCR Primers used in the present study

Primer name	Sequence
LST_NdeI_fw	ACTACATATGGCAGCAACCCATGAAC
LST_SacI_rv	TGATGAGCTCTTTGATGGTGCCC
LST_BamHI_rv	GCCGGATCCTTATTTGATGGTGC
CHAPGH15_NdeI_fw	TACCACATATGGCCAAAACACAGGC
CHAPGH15_SacI_rv	TGATGAGCTCTTTGATTGTACCCCAC
LysK_LST_NdeI_fw	ATACATATGGCTAAGACTCAA
LysK_LST_BamHI_rv	ATACTGGATCCTTACTTTATAGTTCCCCACAG
LysK_LST_SacI_rv	AATCGAGCTCCTTTATAGTTCCCCACAG
CHAPTw_NdeI_fw	ATGACATATGAAAACGCTGAAACAAGCG
CHAPTw_SacI_rv	AACTGAGCTCTTTGATTTCACCCCAC
CHAPK_Nde1_fw	ATACATATGGCTAAGACTCAA
CHAPK_SacI_rv	AATCGAGCTCCTTTATAGTTCCCCACAG
CHAPK_BamHI_rv	GCCGGATCCTTACTTTATAGT
LysK_NdeI_fw	ATACATATGGCTAAGACTCAA
LysK_BamHI_rv	GCCGGATCCTTATTTGAATACTCCCC
LysK_SacI_rv	CGACCGAGCTCTTTGAATACTCCC
CHAPSEP_NdeI_fw	GGAGATATACATATGAAAACCAAAAAACAGGCC
CHAPSEP_SacI_rv	CCAGAGCTCTTTGATCTCACCCC
