# Supplementary material for: Targeting Hidden Pathogens: Cell-Penetrating Enzybiotics Eradicate Intracellular Drug-Resistant Staphylococcus aureus
Source: mBio. 2020 Apr 14;11(2):e00209-20. doi: 10.1128/mBio.00209-20 (PMC7157818; doi:10.1128/mBio.00209-20)
Supplement: TABLE S4 [file mBio.00209-20-st004.pdf]

TABLE S4 Plasmids used and created in the present study

Construct Name	Source
pET302_LST	this study
pET302_CHAPTw_SH3b2638	laboratory collection
pET302_CHAPGH15_SH3bALE1	laboratory collection
pET302_CHAPK_AmiK_SH3bLST	this study
pET302_LysK_LST	this study
pET302_CHAPSEP(L)_SH3b2638	laboratory collection
pET302_LysK	this study
pET302_LST_TAT	this study
pET302_CHAPTw_SH3b2638_TAT	this study
pET302_CHAPGH15_SH3bALE1_TAT	this study
pET302_CHAPK_AmiK_SH3bLST_TAT	this study
pET302_LysK_LST_TAT	this study
pET302_LysK_TAT	this study
pET302_CHAPSEP(L)_SH3b2638_TAT	this study
pET302_LST_Penetratin	this study
pET302_CHAPTw_SH3b2638_Penetratin	this study
pET302_CHAPGH15_SH3bALE1_Penetratin	this study
pET302_CHAPK_AmiK_SH3bLST_Penetratin	this study
pET302_LysK_LST_Penetratin	this study
pET302_CHAPSEP(L)_SH3b2638_Penetratin	this study
pET302_LysK_Penetratin	this study
pET302_LST_KalaSyn	this study
pET302_CHAPTw_SH3b2638_KalaSyn	this study
pET302_CHAPGH15_SH3bALE1_KalaSyn	this study
pET302_CHAPK_AmiK_SH3bLST_KalaSyn	this study
pET302_LysK_LST_KalaSyn	this study
pET302_LST_Pvec	this study
pET302_CHAPTw_SH3b2638_Pvec	this study
pET302_CHAPGH15_SH3bALE1_Pvec	this study
pET302_CHAPK_AmiK_SH3bLST_Pvec	this study
pET302_CHAPSEP(L)_SH3b2638_Pvec	this study
pET302_LysK_LST_Pvec	this study
pET302_LysK_Pvec	this study
pET302_CHAPSEP(L)_SH3b2638_Phylomer1	this study
pET302_LysK_Phylomer1	this study
pET302_CHAPSEP(L)_SH3b2638_Phylomer2	this study
pET302_LysK_Phylomer2	this study
pQE30Xa_H_Xa_CHAPTw_CHAPTw_Sh3bLST[1]	laboratory collection
pQE30Xa_H_Xa_CHAPTw_CHAPTw_SH3b2638a[1]	laboratory collection
pQE30_H_CHAPTw_AmiTw_SH3bTw[1]	laboratory collection
pQE30_H_CHAPTw_Ami2638a_M23LST_SH3b2638a[1]	laboratory collection

pQE30_H_LST[1]	laboratory collection
pET302_M23LST(L)_SH3b2638a	laboratory collection
pET302_M23LST_SH3bLST_M23LST	laboratory collection
pQE30_H_M23LST_M23LST_M23LST_SH3bLST[1]	laboratory collection
pET21a_LST_PTD9_H[1]	laboratory collection
pQE30Xa_H_Xa_LST[1]	laboratory collection
pQE30Xa_H_Xa_M23LST_CBD2638a[1]	laboratory collection
pQE30_H_LST_LST[1]	laboratory collection
pQE30_H_TEV_M23LST_SH3bLST[1]	laboratory collection
pET21a_LST_PTD7_H[1]	laboratory collection
pQE30_H_TEV_M23LST_M23LST_SH3bLST[1]	laboratory collection
pQE30Xa_H_Xa_M23LST_M23LST_Sh3bLST[1]	laboratory collection
pET21a_LST_H[1]	laboratory collection
pET21a_LST_PTD10_H[1]	laboratory collection
pET21a_LST_PTD8_H[1]	laboratory collection
pET21a_M23LST_CHAPLysH5_AmiLysH5_Sh3bLST[1]	laboratory collection
pET302_M23LST_Ami2638a_SH3b2638a	laboratory collection
pET21a_CHAPφ11_Amiφ11_M23LST_SH3bLST[1]	laboratory collection
pET21a_PepλSA2_(GSL0408)_CHAPK_M23LST_SH3bLST_H[1]	laboratory collection
pET21a_PepλSA2_CHAPK_M23LST_SH3bLST_H[1]	laboratory collection
pET_21a_LysK_LST_H[1]	laboratory collection
pQE30_H_TEV_CHAPK_CHAPK_SH3bLST[1]	laboratory collection
pET21_LysK_H[1]	laboratory collection
pQE30_H_TEV_CHAPK_SH3bLST[1]	laboratory collection
pET21a_CHAPK_SH3bLST_PTD3_H[1]	laboratory collection
pET21a_CHAPK_AmiK_SH3bLST_H[1]	laboratory collection
pET21a_CHAPK_SH3bLST_H[1]	laboratory collection
pQE30_H_LysK[1]	laboratory collection

[1] Construct containing a 6xHis tag
